# Natural variation in expression of genes associated with carotenoid biosynthesis and accumulation in cassava (*Manihot esculenta* Crantz) storage root

**DOI:** 10.1186/s12870-016-0826-0

**Published:** 2016-06-10

**Authors:** Luiz JCB Carvalho, Marco AV Agustini, James V Anderson, Eduardo A Vieira, Claudia RB de Souza, Songbi Chen, Barbara A Schaal, Joseane P Silva

**Affiliations:** EMBRAPA Genetic Resources and Biotechnology, Brasília, DF Brazil; USDA-ARS, Sunflower and Plant Biology Research Unit, Fargo, ND USA; EMBRAPA Cerrados, Brasília, DF Brazil; Universidade Federal do Para, Belém, Pará Brazil; Tropical Crops Genetic Resources Institute, Chinese Academy of Tropical Agriculture, Hainan, China; Department of Biology, Washington University in St. Louis, St Louis, MO USA

**Keywords:** Cassava storage root, Carotenoid, β-carotene, HPLC-DAD, Microarray, qRT_PCR

## Abstract

**Background:**

Cassava (*Manihot esculenta* Crantz) storage root provides a staple food source for millions of people worldwide. Increasing the carotenoid content in storage root of cassava could provide improved nutritional and health benefits. Because carotenoid accumulation has been associated with storage root color, this study characterized carotenoid profiles, and abundance of key transcripts associated with carotenoid biosynthesis, from 23 landraces of cassava storage root ranging in color from white-to-yellow-to-pink. This study provides important information to plant breeding programs aimed at improving cassava storage root nutritional quality.

**Results:**

Among the 23 landraces, five carotenoid types were detected in storage root with white color, while carotenoid types ranged from 1 to 21 in storage root with pink and yellow color. The majority of storage root in these landraces ranged in color from pale-to-intense yellow. In this color group, total β-carotene, containing *all*-*E*-, 9-*Z*-, and 13-*Z*-β-carotene isomers, was the major carotenoid type detected, varying from 26.13 to 76.72 %. Although no α-carotene was observed, variable amounts of a α-ring derived xanthophyll, lutein, was detected; with greater accumulation of α-ring xanthophylls than of β-ring xanthophyll. Lycopene was detected in a landrace (Cas51) with pink color storage root, but it was not detected in storage root with yellow color. Based on microarray and qRT-PCR analyses, abundance of transcripts coding for enzymes involved in carotenoid biosynthesis were consistent with carotenoid composition determined by contrasting HPLC-Diode Array profiles from storage root of landraces IAC12, Cas64, and Cas51. Abundance of transcripts encoding for proteins regulating plastid division were also consistent with the observed differences in total β-carotene accumulation.

**Conclusions:**

Among the 23 cassava landraces with varying storage root color and diverse carotenoid types and profiles, landrace Cas51 (pink color storage root) had low *LYCb* transcript abundance, whereas landrace Cas64 (intense yellow storage root) had decreased *HYb* transcript abundance. These results may explain the increased amounts of lycopene and total β-carotene observed in landraces Cas51 and Cas64, respectively. Overall, total carotenoid content in cassava storage root of color class representatives were associated with spatial patterns of secondary growth, color, and abundance of transcripts linked to plastid division. Finally, a partial carotenoid biosynthesis pathway is proposed.

**Electronic supplementary material:**

The online version of this article (doi:10.1186/s12870-016-0826-0) contains supplementary material, which is available to authorized users.

## Background

Carotenoids are a family of C40 isoprenoid pigments including approximately 600 identified structures in higher plants. The accumulation of intermediary carotenoids and their stable natural isomers (Z_*iso*) varies in accordance with plant species and plant organ types. A unified super pathway of carotenoid biosynthesis for cassava has been proposed at Plant Metabolic Network (PMN) online databases.

Critical functions of carotenoids in plants include: harvesting light during photosynthesis [1]; providing flowers and fruits with animal attracting color that facilitate pollination and seed dispersal [2]; stabilizing membrane lipids via antioxidant properties [3]; as well as providing cleavage products (ABA, strigolactones, β-cyclocitral) that act as signal molecules for regulation of physiological functions under abiotic stress [3], modulating developmental processes [4] and plant environmental responses [5]. Carotenoids are also viewed as producers of exudates (Strigolactones) in mycorrhizal fungi root symbiosis [6] affecting different hormonal pathways associated with lateral root formation and root-hair elongation. In addition, carotenoids serve as precursors of vitamin A, which is one of the most important micronutrients affecting human health [7, 8]. Diets containing carotenoid-rich vegetables, fruits, and roots can protect against some cancers, heart disease, cataracts, and ultraviolet-induced skin damage [9]. However, in spite of what is known about carotenoids, some essential information is still lacking. For example, their function in underground organs like cassava storage root (CSR), as well as the molecular genetics and mechanisms responsible for their massive accumulation in parenchyma cells as secondary growth proceeds, especially in CSR.

Biochemical characterization of enzymes responsible for carotenoid biosynthesis has been hampered by difficulties in purifying active enzyme forms. Many of these enzymes are membrane-associated proteins, which impedes enzyme activity assays [10, 11]. However, approaches such as color complementation in *E. coli* and molecular identification of mutants [11] have been helpful in understanding relationships between expression of genes associated with carotenoid biosynthesis and accumulation of carotenoids in plants. Although all structural genes involved in plant carotenoid syntheses are known, information on how differential expression of these structural genes affects carotenoid content or composition in underground storage organs is incomplete. At least in underground organs like cassava storage root, there is not enough information to draw a conclusion on regulation of synthesis and sequestration. Thus, molecular and biochemical research related to accumulation of carotenoids in CSR could serve as an important biological system for studying carotenoid metabolism, in addition to improving the nutritional value of CSR [12–16].

At the molecular level, steps in the carotenoid biosynthesis pathway in plants are well characterized [11, 17, 18], including processes associated with condensation, oxygen desaturation, isomerization, cyclization, oxygenation, and hydroxylation. The first committed step in the biosynthesis of carotenoids is the head-to-head condensation of two molecules of geranylgeranyl pyrophosphate to form the colorless intermediate phytoene. This reaction is catalyzed by the enzyme phytoene synthase (PSY), which is regarded as the rate-limiting step in some plant systems. In plants, the enzymes phytoene desaturase (PDS) and ζ-carotene desaturase (ZCD), each introduce two symmetric double bonds that, in parallel with carotenoid isomerase (CRTISO), form all-*E*-lycopene. Subsequently, the ends of the linear carotenoid (lycopene) are cyclized by β-lycopene cyclase (LYCb) and ε-lycopene cyclase (LYCe), forming the xanthophylls and the introduction of various oxygen functions by ε-ring hydroxylase (BCHe), β-ring hydroxylase (BCHb), zeaxanthin epoxidase (ZEP), neoxanthin synthase (NXS), and violaxanthin de-epoxidase (VDE).

While there is diversity in CSR color, resulting from the accumulated carotenoids, the developmental and regulatory roles of carotenoids related to storage root formation are unknown. Thus, studies to elucidate the carotenoid profiles from CSR with diverse color may provide new insights on unknown functions of carotenoids in this non-green organ. In addition, the diversity in color observed in cassava landraces [19] could contribute to a human diet that combines macronutrients (starch) and micronutrients (β-carotene, lutein and lycopene) in the same source of staple food. To take advantage of the existing genetic resources regarding the agro biodiversity of cassava in the Amazon region, its origin and center of domestication [19, 20], the primary focus of this study was four fold: (1) to identify natural genetic variations in the carotenoid synthesis pathway that could be of importance in elucidating the functions of carotenoids in non-green tissues, (2) to identify variations in carotenoid content and types in relation to tissue age of the CSR, (3) to identify differences in expression of genes associated with carotenoid synthesis and accumulation, and (4) propose a natural carotenoid biosynthesis pathway for CSR.

## Methods

### Ethics statement

A germplasm collection of cassava plants is maintained in the Germplasm Base Collection (COLBASE) of EMBRPA Genetic Resources and Biotechnology. To access diversity in carotenoid composition of cassava storage root, experiments were performed under a license from the Genetic Heritage Management Council (CGEN) as required [21] and follow the approval from the local Ethical Review Panel of EMBRAPA Genetic Resources and Biotechnology [22].

### Plant material and tissue preparation

#### Plant material

Cassava plants used in this study are representative of the diverse range of storage root (SR) color among genetic stocks obtained from landraces collected in the Amazon (Additional file 1: Figure S1, Panel A). A set of 23 landraces (Additional file 2: Table S1) cultivated in field plots at EMBRAPA Genetic Resources and Biotechnology, representing individuals from 5 color classes, were processed separately three times.

#### Tissue preparation

Storage roots that were 30–40 cm long and 3–4 cm in diameter were washed with tap water, peeled to a length of 10 cm, containing the central part, and manually sliced. The tissue was immediately frozen in liquid nitrogen, freeze dried, ground into powder with mortar and pestle, and stored at −80 °C prior to use for carotenoid separation, identification, and quantification by HPLC-DAD. Additionally, fresh tissue sampling layers were prepared from storage roots as illustrated in Additional file 1: Figure S1, Panel B. For total RNA extraction, fresh and intact storage roots were peeled and processed immediately after harvest.

### Carotenoid extraction and quantification

#### Carotenoid extraction

Between 5 and 20 mg of powder, depending on the intensity of CSR color, was transferred to a mortar and hydrated. A scoop of Celite powder was added and the mixture crushed with acetone and filtered through a sintered glass funnel. Following three washes with acetone, a sequential transfer of 1/5 of the acetone extract volume was partitioned to 50 mL petroleum ether in a separatory funnel. Slowly, 300 mL of ddH_2_O was added, allowing the two phases to separate.

The lower aqueous-acetone phase was discarded by washing five times with ddH_2_O. The solvent phase was collected in a volumetric flask after having passed through anhydrous sodium sulfate. This procedure was employed to generate HPLC-DAD profiles and for quantification of specific carotenoids. An alternative extraction procedure was applied for separate samples used for carotenoid quantification by spectrophotometer. This procedure includes hydration of the sample (100 to 2000 mg powder) with ddH_2_O, addition of petroleum ether followed by four to six pulses with a Polytron, centrifugation (4000 rpm, 4 °C, 20 min), sonication to disperse any micelles formed, and collection of the solvent phase. This was then filtered through anhydrous sodium sulfate and its volume adjusted with petroleum ether until λmax and maximum absorbance for each sample at 300 to 600 nm were obtained. A total of 23 landraces were processed for different analytical procedures, including total carotenoid content and HPLC-DAD analyses depend on the question addressed in particular experiments.

#### Total carotenoid quantification by spectrophotometer

Total carotenoid distribution in tissue sample layers was estimated according to (μg/g = (Integrated area at OD_445_ read for plant extract/Integrated area at OD_445_ read for standard β_carotene) [23]. Optical density readings were taken using SPECTRA_Max (Model 384 PLUS) Spectrophotometer.

#### Carotenoids separation, identification and quantification by HPLC-DAD

Carotenoid standard preparation, separation and identification used for standard curves was carried out as previously described [23, 24]. Landrace reference profiles are available for reads at visible light (Additional file 3: Figure S2) wavelength at λmax = 455 nm) and for reads at UV light (Additional file 4: Figure S3, wavelength at λmax = 370 nm). Similarly, a set of 23 landraces were used for specific carotenoid identification and quantification by integrating the peak area in the HPLC-DAD chromatogram in both λmax of each carotenoid. A correction factor for specific carotenoid extinction coefficient [17, 18, 23, 24] was derived in relation to the extinction coefficient of β-carotene used in the standard curve. For those carotenoids with unknown extinction coefficient, this ratio was considered as 1 as recommended [23, 24]. The corrected areas for specific peaks were used for calculation using a standard curve prepared with β-carotene extracted from carrot as previously described [23, 24] and expressed in terms of μg/g DWt. The HPLC-DAD chromatograms for the 23 landraces studied are showed in Additional file 5: Figure S4, Additional file 6: Figure S5, and Additional file 7: Figure S6 for reporting conveniences of graphic quality.

### Cloning and sequencing of genes associated with the carotenoid biosynthesis pathway

#### RNA extraction and cDNA cloning of genes coding for carotenoid biosynthesis enzymes

Total RNA was extracted from Cas31 as previously described [25] and cDNA was cloned for subsequent sequencing of genes coding for enzymes involved in carotenoid biosynthesis. Fresh tissues (5 g) of storage roots were ground to powder in liquid nitrogen, and 20 mL of RNA extraction buffer (100 mM NaCl, 10 mM Tris–HCl pH 7.5, 1 mM EDTA and 1 % SDS) as well as 20 mL phenol:chloroform:isoamyl alcohol (25:24:1) were added. The mixture was vortexed, incubated for 10 min at room temperature, and then centrifuged (7000 rpm, 4 °C, 50 min). The supernatant was collected and nucleic acid was precipitated by adding 1/10 volume of 3 M sodium acetate and 2.5 volume of cold ethanol. After incubation at −20 °C for 4 h, the sample was centrifuged (7000 rpm, 4 °C, 3 min) and the pellet was re-suspended in ddH_2_O. A separate RNA precipitation was carried out by adding an equal volume of lithium chloride (4 M) and incubating the sample overnight at 4 °C. The RNA pellet was collected by centrifugation (10,000 rpm, 4 °C, 20 min). After the pellet had been re-suspended in ddH_2_O, RNA was precipitated with sodium acetate, cold ethanol and centrifugation as described above. The resulting pellet was re-suspended in 800 μL ddH_2_O, and total RNA was quantified by a spectrophotometer standard procedure and stored at −80 °C for further use.

Single strand cDNA was synthesized with M-MLV reverse transcriptase, total RNA, and oligo-dT primers (Invitrogen procedure). Double-strand cDNA was amplified by PCR, using a cycling program of 1 cycle at 94 °C (5 min), 35 cycles at 94, 45, and 72 °C, and extension at 72 °C. Initially, specific gene sequences of *Arabidopsis thaliana* were used to generate primers for the PCR amplification step in the preparation of cDNA fragments to be cloned. Single bands of amplified products were separated in an agarose gel (1 %) and purified using the QIAquick Gel Extraction Kit according to manufacturer (Qiagen), precipitated with 2 volumes of cold ethanol, centrifuged (12,000 rpm, 20 min, 4 °C), and re-suspended in 3 μL of ddH_2_O. The PCR product was inserted into pGEM®-T Easy Vector system according to manufacture (Promega) with overnight incubation. Transformation was performed by electroporation, following the addition of 3 μL of ligated vector and insert to 50 μL of XL1-blue competent cells with resistance to kanamycin. Ten single blue colonies were selected, multiplied in LB medium and used for sequencing.

#### cDNA sequencing and analysis

Colony plaques were prepared and single bacterial colonies were transferred to 96-well microtiter plates containing LB and ampicillin (100 mg/L) and allowed to grow overnight by shaking at 37 °C (18 h at 320 rpm). The plasmid DNA was purified by standard alkaline lysis method with one modification at the end of the procedure, where the supernatant was passed through a multi-screen filter (Millipore) prior to DNA precipitation. The purified DNA was resuspended in autoclaved milliq-H_2_O. The sequencing reactions were performed with 200 ng of DNA by standard protocols of the ThermoSequenase II Dye Terminator Cycle Sequencing Kit (Amersham-Pharmacia Biotech) using universal M13 forward and reverse primers and the capillary sequencer MegaBACE 1000. The samples were electro injected with 2KV for 100 s, and the fragments were separated at 9 kV for 100 min. The electropherograms were submitted to the Phred, Phrap, and Consed package for sequence quality evaluation. The sequences with Phred > 20 were registered and used for BLAST analysis against the NCBI GenBank database [26], and annotated to the cassava genome [27] (Additional file 8: Table S2). Primers were designed from cDNA fragment sequences (Additional file 9: Table S3) of annotated proteins to be used for qRT_PCR gene expression analysis.

### Global transcript profiling by microarray analysis

#### RNA isolation, quantification and microarray assay analysis

Total RNA, from tissue layer 3 was extracted twice in independent events, isolated by phenol-chloroform procedure as described [25], and treated with RNAse-free DNAse. Total RNA was quantified using the QuantiT™ RiboGreen® RNA Kit according to the manufacturer protocol (Molecular Probe). The microarray analysis followed the experimental design of Loop Dye Swap hybridization system, three biological replications, three sample replications, two technical replications, and the dye replication as described earlier [28–30]. Labeling of cDNA and chip hybridization were accomplished using a kit from Invitrogen (Platinum® PCR SuperMix), following the procedure recommended. Total RNA (30 μg) was used to prepare cDNA probes labeled with Cy3 and Cy5. Data were transferred to EXCEL spread sheets, and deposited in our domestic gene expression data base for cassava storage root at EMBRAPA Genetic Resources and Biotechnology (Brasilia, DF. Brazil).

#### Data analysis

A data set for gene expression analysis workflow considered image and data quality evaluation at Gene Pix Pro software https://www.moleculardevices.com/ using array design, image quality (background, intensity & reproducibility), spot quality (center location, background, intensity, noise, specificity, morphology & reproducibility), and spike controls to determine transcript abundance. High quality data was processed (normalized), and identification of differentially expressed genes (DEG at *p* < 0.05) were identified using GeneMath software http://www.applied-maths.com/applications. Identified transcript sequences including the cloned genes (cDNA fragments) coding for the six major enzymes used for the qRT_PCR assay, the microarray assay for genes coding for enzymes in the pathway and the plastid multiplication hybridization assay were BLASTED to the cassava genome at Phytozome [27] as showed in Additional file 8: Table S2. Identified genes coding for enzymes related to carotenoid biosynthesis were used to locate their association within the particular step in the cassava supper pathway at Plant Metabolic Network (PMN) [31]. The intermediates in this reference pathway were confirmed for its identified presence in the carotenoid HPLC_DAD profile of landrace Cas64 (containing 19 identified carotenoids in its intense yellow color SR) and landrace Cas51 (containing a single peak in its pink color SR). These information were used for the recognition of substrate and product for each step in the supper pathway proposed at PMN http://pmn.plantcyc.org/CASSAVA/NEW-IMAGE?type=PATHWAY&object=CAROTENOID-PWY based on The Arabidopsis Information Resource (TAIR), identification of the particular enzyme name, enzyme code, and predicted enzyme reaction (Table 1). Finally statistical analysis was performed for correlation studies and non-parametric statistical tests by using the R_statistics https://cran.r-project.org/. This analysis allowed us to propose a specific diagram for the carotenoid biosynthesis pathway of yellow CSR (Cas64) incorporated with the pink color CSR (Cas51). Microarray data values were normalized (Ln) and gene expression evaluated in relation to that by qRT_PCR for the six major enzymes (PSY, PDS, CRTISO, BCHb, LYCb, NXS) by correspondence analysis [32] using the R_statistics (http://www.R-project.org/.) procedure [33], which incorporated the four landraces with major contrasting HPLC_DAD carotenoid profiles.

**Table 1:** Spectroscopic characteristics of carotenoid identity

Peak #	Retention time (minute)	Carotenoid #	Carotenoid name	λ_max1_	λ_max2 (II)_	λ_max3 (III)_	Percentage (% III/II)
01	4.8	01	Neoxanthin	416	440	470	80
02	4.9	02	Violaxanthin	418	443	472	74
03	5.0	03	Zeaxanthin	423	442	471	40
04	5.3	04	Crocetin	399	422	448	61
05	7.9	05	Lutein	422	445	473	60
06	8.0	06	Antheroxanthin	425	445	474	15
07	17.2	07	Lycopene	446	473	504	98
08	18.5	08	β-cryptoxanthin	422	442	470	92
09	24.1	09	α-Zeacarotene	402	422	448	22
10	25.7	10	Neurosporene	418	442	470	71
11	28	11	ζ carotene	399	423	449	92
12	34.5	12	β-zeacarotene	399	423	449	69
13	41.6	13	*All trans* β-carotene	424	446	479	10
13	41.6	14	Phytofluen1	349	388	388	79
14	43.02	15	*9-cis*-β-carotene	431	445	472	78
14	43.02	16	Phytofluen2	349	388	447	78
15	45.7	17	*13-cis*-β-carotene	424	446	473	19
15	45.7	18	Phytofluen 3	279	341	447	19
16	52.3	19	Phytoene	275	286	299	12

Spectroscopic characteristics of 19 carotenoid types obtained from HPLC_DAD for landrace Cas64, as reference profile, plus Cas51 for carotenoid type identification in the original chromatograms of 23 landraces studied. HPLC_DAD profiles were obtained in C18 Waters Spherisorb ODS_2 (4.5 × 150mm. 5 mm) column using mobile phase Methanol:Ethylacetate:Acetonitrile (1:1:8) in a isocratic run for 60 min. For qualitative analysis the chromatograms were read at 455 nm for color and at 350 nm for colorless carotenoid types. Carotenoid types were identified by comparison with purified samples for β-carotene from carrot, Lutein from capuchin flower (*Tropaeolum majus*), and Lycopene from tomato fruit. As a way to reduce misidentification of other unknown carotenoids, minimum identification criteria as previously described [10.21.22], was performed and included comparisons of retention time in minutes (RT), peak number (P#) in the HPLC_DAD profile, UV-visible photodiode array spectra, lambda maximum (λmax^1^. λmax^2^ (III). λmax^3^ (II) for each carotenoid in acetonitrile solvent, and fine structure defined as %III/II

### Quantification of transcripts associated with carotenoid biosynthesis by qRT-PCR

#### RNA isolation, quantification, and cDNA synthesis

Total RNA was extracted twice as described above in independent events and isolated by a phenol-chloroform procedure as described above and treated with RNAse-free DNAse. Total RNA was quantified using the Quant-iT™ RiboGreen® RNA Kit according to the manufacturer (Molecular Probe). Estimation of transcript levels for each corresponding gene sequence was performed by quantitative real-time PCR (qRT-PCR). Total RNA (2.8 μg) was reverse transcribed in a 20 μL reaction volume using SuperScript® III Platinum® Two-Step qRT-PCR Kit (Invitrogen) for each extraction. Parallel reactions for each extraction were performed without SuperScript® III first step (RT control) to assess potential contamination of genomic DNA in the extractions. The reactions were terminated by heat inactivation at 70 °C for 15 min. Subsequently, the cDNA products were treated with 2 units of RNase H for 20 min at 37 °C, then diluted in autoclaved mqH_2_O to 20 ng/μL and stored at −20 °C.

#### Gene-specific primers, certified standard and housekeeping gene primers

Fluorogenic primers (FAM labeled LUX primer) and corresponding unlabeled primers were designed using the LUX Designer-Desktop version (Invitrogen) for each sequence of the cDNA fragment coding for carotenoid biosynthesis genes. Standard certified primers for 18S ribosomal (Invitrogen Cat. 115HM-02) Gus (Invitrogen Cat. 112H-02) and qPCR plasmid standards (Invitrogen Cat. 11741-100) with Gus ORF were used as internal control housekeeping genes and quantitative standards to generate a standard curve, respectively. All primers were synthesized and purchased from Invitrogen.

#### Quantitative real time PCR amplification

qRT-PCR assays were performed in triplicate for each extraction on a Bio-Rad system (BioRad model iCycler) using 18S ribosomal RNA as an internal control reaction. PCR efficiency evaluations and GUS certified primers were used to obtain a quantitative standard curve for absolute expression analysis of target transcript level in samples. Of the diluted cDNA, 1.5 μL (30 ng) was used as a template in a 25 μL PCR reaction containing 1× platinum quantitative PCR SuperMix-UDG, 0.15 μM of non-fluorogenic and 0.3 μM of LUX fluorogenic primer. The PCR thermal-cycling parameters were 50 °C for 2 min, 95 °C for 2 min, followed by 40 cycles of 95 °C for 10 s, 50 °C for 30 s, and 72 °C. For each experiment at least three replicates were used.

### Data analysis

In order to access the level of each transcript corresponding to target genes in all samples, the qRT-PCR amplification procedure was designed to use quantification expression using μg of total RNA as normalizer. PCR efficiency was accessed by certified quantitative plasmid standards, internal controls for housekeeping genes, and negative control for detection of carry over contaminant DNA from the extraction procedure. By setting up this design and conditions, a quantitative standard curve for a dilution series covering a range of 3.34 × 10^−5^ , 3.34 × 10^−4^, 3,2 × 10^−3^, 3.34 × 10^−2^ 3.34 × 10^−1^, 3.34 × 10, and 3.34 × 10^1^ nanomole for the 757 reference plasmid was obtained according to qRT-PCR plasmid standard kit supplier (Invitrogen). Preliminary experiments with two unknown samples were performed to set up optimal qRT-PCR amplification conditions such as reliable exponential phase of amplification, qRT-PCR efficiency ranging from 90 to 105 %, defining the Ct threshold, and setting up baseline and evaluation of each primer set for target genes of the unknown samples. After setting up the optimized conditions for Ct values for all unknown samples and replications, they were interpolated from the standard curve with the logarithm of the initial value of the standard plotted along the x-axis and their corresponding Ct value along the y-axis. The equation for linear regression line (y = −3.316x + 22.329; *R* = 0.991) was used for estimating the level of transcript in the unknown smples. Raw data were transferred to an EXCEL spread sheet and data were analyzed using the statistical language program R-Statistics free to download at https://cran.r-project.org/ [33] to determine effect of representative landrace on the expression of genes measured either by microarray or qRT_PCR technologies. First, conventional ANOVA was performed to check for the normal distribution assumption as well as variance heterogeneity through graphical analysis and formal tests [34, 35]. As the results indicated violation of the basic ANOVA assumptions, we adopted the nonparametric Kruskal-Wallis test from *agricolae* library [36]. Equal approach and procedures were used for other traits thought-out the current manuscript.

## Results

### Identification and content of carotenoids in storage root of cassava landraces

#### Separation, detection, and identification of carotenoids by HPLC-DAD

Analysis of crude, non-polar extracts from CSR by HPLC-DAD [23, 24] identified a total of 19 carotenoids types (Table 2), which includes three β-carotene isomers (*All_trans, 9_cis,13_cis*) and three forms of Phytofluene (Phytoene1, Phytoene 2, and Phytoene3).

**Table 2:** Carotenoid composition and content

Color classes	RT	IY	IY	IY	IY	IY	IY	IY	IY	IY	Y	Y	Y	Y	Y	Y	Py	PY	PY	PY	PY	PY	W	P
Carotenoid type		Cas64	Cas56	Cas31	Cas61	Cas32	Cas57	Cas47	Cas62	Cas34	Cas68	Cas52	Cas35	Cas33	Cas60	Cas53	Cas66	Cas30	Cas74	Cas70	Cas71	Cas37	IAC12	Cas51
Neoxanthin	4.8	1.4 (±0.13)	0.8 (0.32)	1.37 (±0.07)	0.35 (±0.07)	0.83 (±0.07)	0.15 (±0.02)	1.43 (±0.07)	0	0.86 (±0.11)	0.39 (±0.05)	0.76 (±0.09)	0.46 (±0.04)	0.42 (±0.06)	0.18 (±0.02)	0.09 (±0.01)	0.05 (0.0)	0.1 (0.01)	0.06 (0)	0.07 (±0.02)	0.28 (±0.03)	0.1 (±0.02)	0.19 (±0.01)	0
Violaxanthin	4.9	1.2 (±0.06)	0.45 (0.22)	0.59 (±0.25)	0.11 (±0.09)	0.22 (±0.07)	0.09 (±0.01)	0.52 (±0.029)	0	0.25 (±0.16)	0.2 (±0.07)	0.19 (±0.05)	0.18 (±0.06)	0.14 (±0.02)	0.07 (±0.03)	0	0.03 (0.0	0	0	0	0.07 (±0.01)	0	0.02 (0)	0
Zeaxanthin	5.0	0	1.65 (0.20)	1.52 (±0.12)	0.51 (±0.04)	0	0	2.06 (±0.011)	0	1.05 (±0.15)	0.51 (±0.06)	0.41 (±0.10)	0	0.22 (±0.04).	0	0	0.05 (0.0)	0	0	0	0	0	0	0
Crocetin	5.3	1.7 (±0.04)	0.69 (0.29)	1.16 (±0.01)	0	0.18 (±0.02)	0	0.10 (±0.02)	0	0.83 (±0.08)	0.43 (±0.08)	0.19 (±0.02)	0.71 (±0.04)	0	0.09 (±0.04)	0.19 (±0.01)	0.04 (0)	0.18 (0.02)	0.04 (0)	0.07 (±0.04)	0.06 (0)	0	0.3 (±0.03)	0
Lutein	7.9	3.6 (±0.11)	6.18 90.06)	2.16 (±0.10)	0.27 (±0.04)	0.35 (±0.02)	0.46 (±0.07)	2.31 (±0.17)	0	2.43 (±0.22)	1.57 (±0.16)	0.61 (±0.06)	0.94 (±0.03)	0	0.15 (±0.01)	0.5 (±0.01)	0.05 (0)	0.14 (0.03)	0.09 (0)	0.12 (±0.02)	0.12 (±0.02)	0.04 (0)	0.3 (±0.02)	0
Anteroxanthin	8.0	0	0	0	0	0	0	0.89 (±0.04)	0	0	0	0	0	0	0	0	0	0	0	0	0	0	0	0
Lycopene	17.2	0	0	0	0	0	0	0	0	0	0	0	0	0	0	0	0	0	0	0	0	0	0	14.84 (±0.07)
β-cryptoxanthin	18.5	0.6 (±0.04)	0.52 (0.25)	0.91 (±0.04)	0.11 (±0.09)	0.32 (±0.04)	0	0.73 (±0.08)	1.56 (±0.12)	0	0	0	0	0	0	0	0	0	0	0	0	0	0	0
β-zeacaroteno	24.1	0	0	0	0	0	0	0	0.48 (±0.11)	0	0	0	0	0	0	0	0	0.05 (0)	0	0	0	0	0	0
Neurosporene	25.7	1.1 (±0.32)	0.83 (0.42)	0	0	0	0	0.68 (±0.07)	1.34 (±0.03)	0.76 (±0.05)	0	0	0.19 (±0.02)	0	0	0	0	0	0	0	0	0	0	0
ε − carotene	28	0.6 (±0.03)	0	0	0	0	0	0	1.47 (±0.09)	0	0	0	0	0	0	0	0	0	0	0	0	0	0	0
ζ-zeacarotene	34.5	0	0	0	0	2.61 (±0.09)	0	0	2.03 (±0.09)	0	0	0	0	0	0	0	0	0	0	0	0	0	0	0
*All trans* β − carotene	41.6	6.3 (±0.23)	5.16 (0.04)	5.71 (±0.26)	0.88 (±0.05)	1.54 (±0.09)	0.62 (±0.10)	7.23 (±0.38)	17.84 (±0.11)	6.24 (±0.17)	1.21 (±0.08)	1.6 (±0.03)	0.9 (±0.09)	0	0	0	0	0	0.13	0.24 (±0.003)	0.89 (±0.05)	0	0	0
Phytofluene1	41.6	0	3.65 (0.51)	0	0.54 (±0.05)	0	2.37 (±0.04)	5.11 (±0.08)	0	0	0	0	0	0.5 (±0.01)	0	0	0.06 (0)	0	0	0	0	0.9 (±0.12)	0.9 (±0.09)	0
9-cis − β − carotene	43.0	5.8 (±1.35)	4.04 (0.24)	4.28 (±0.59)	0.59 (±0.05)	1.06 (±0.02)	0.45 (±0.19)	5.57 (±0.42)	10.58 (±0.27)	5.89 (±0.10)	0.88 (±0.04)	1.34 (±0.11)	0.86 (±0.06)	0.54 (±0.02)	0.6 (0.04)	0.67 (±0.02)	0.07 (0)	0.41	0.09	0.15 (±0.02)	0.52 (±0.04)	0.6 (±0.13)	0.3 (±0.02)	0
Phytofluen2	43.0	0	0.38 (0.16)	0	0.04 (±0.03)	0	0.26 (±0.04)	0.51 (±0.013)	0	0.52 (±0.20)	0.14 (±0.03)	0	0.83 (±0.05)	0.07 (±0.02)	0	0.6 (±0.05)	0.03 (0)	0	0	0	0	0	0	0
13-cis-β-carotene	45.7	2.0 ± 0.12)	1.62 (0.13)	2.29 (±0.23)	0.23 (±0.02)	0	0.20 (±0.07)	2.22 (±0.26)	5 (±0.26)	1.96 (±0.26)	0.52 (±0.04)	0.52 (±0.14)	0.26 (±0.06)	0.29 (±0.01)	0.38 (±0.01)	0.3 (±0.01)	0.03 (0)	0.26	0.03	0.1 (±0.02)	0.36 (0.0)	0.3 (±0.03)	0.2 (±0.02)	0
Phytofluen3	45.7	0	1.47 (0.08)	0	0.2 (±0.04)	0	1.17 (±0.20)	2.07 (±0.10)	0	1.81 (±0.22)	0.46 (±0.19)	0	0.27 (±0.02)	0.3 (±0.03	0	0.33 (0.02)	0	0	0	0	0	0	0	0
Phytoene	52.3	0	2.27 (0.24)	0	0	0	0	0	15.68 (0.19)	0	0	0	0	0	0	0.93 (0.02)	0	0	0	0	0	0.03 (0)	0.09 (0.01)	0
Total		24.7	29.71	19.9	3.8	7.1	5.8	31.4	55.9	22.6	6.3	5.6	5.6	2.5	1.5	3.6	0.4	1.1	0.4	0.8	2.3	1.9	2.3	14.8
Proportion of β_carotene/total (%)		57.6	36.4	61.4	44.4	36.6	22.0	47.8	59.7	62.3	41.4	61.6	36.1	33.5	66.7	26.9	24.4	58.8	56.8	65.3	76.0	45.8	21.7	0.0

Carotenoid types and content (μg/g DWt.) in storage root of 23 pigmented cassava landraces. Abbreviations accounts for RT = retention time (minutes) of HPLC_DAD in the carotenoid profile. Color class groups were named as IY – Intense yellow. *Y* yellow, *PY* pale yellow, *W* white, *P* pink. Values in parenthesis refer to standard deviation for a particular carotene obtained with two biological replications and calculated from the integration of peak area in the chromatogram

**Fig. 1:**
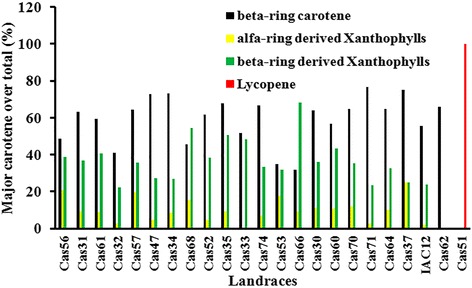
Major carotenoids detected in storage root across the 23 cassava landraces studied. Result refers to proportion (%) of all β-carotene (All, 13Z, 9Z), α-ring xanthophylls, β-ring xanthophylls and lycopene in relation to total carotenoid (μg/gDWt.) as values reported in Table 3 that shows the descriptive statistics obtained with two biological replications

The 19 carotenoid types identified in chromatograms originating from landraces with yellow CSR included three isomer forms of β-carotene. Lutein was widely present across all yellow SR landraces except landrace Cas62, and Cas53. Phytoene was detected in Cas56, Cas62, Cas53, and IAC12, but absent in all the other landraces. *All trans* β-carotene and its isomeric forms, 13-*Z* and 9-*Z*, were the major carotenoid types present in the yellow CSR group (Table 3 and Fig. 1).

**Table 3:** Compiled source of information used for designing a proposed carotenoid biosynthesis pathway diagram for cassava storage root

Enzyme code^a^	Common name enzyme symble	Predicted reaction^b^	cDNA code for microarray spots registered at NCBI^c^	Gene code at NCBI^d^	Gene code at cassava genome^e^	References^f^
EC 2.5.1.32	CasPSY2/partial	2 2-cis,6-trans,10-trans-geranylgeranyl diphosphate < = > 15-cis-phytoene + 2 diphosphate	CV03029A1H10.f1	ADN65331.1	cassava4.1_008121m	This article
EC 2.5.1.32	PSYa	prephytoene diphosphate → 15-cis-phytoene + diphosphate	CV03071B1B02.f1S		cassava4.1_008056m	This article
EC 2.5.1.32	PSY2	2 2-cis,6-trans,10-trans-geranylgeranyl diphosphate < = > 15-cis-phytoene + 2 diphosphate	CV03029A1H10.f1	ACY42665.1	cassava4.1_033101m	Planta 232 (5), 1251–1262 (2010)
EC 1.3.99.29	PDS/partial	15-cis-phytoene + an oxidized electron acceptor < = > all-trans phytofluene + a reduced electron acceptor	CV03049A2F07.f1	ABV01926.1	cassava4.1_004359m	This article
EC 5.2.1.12	CRTISOa	9,15,9′-tri-cis-ζ-carotene < = > 9,9′-di-cis-ζ-carotene	CV03063A1A06.f1		cassava4.1_009948m	This article
EC 5.2.1.13	CasCRTISO/partial	prolycopene < = > all-trans-lycopene		ACI12955.1	cassava4.1_003897m	This article
EC 5.2.1.13	CRTISO	prolycopene < = > all-trans-lycopene	CV01018A2H03.f1		cassava4.1_004361m	This article
EC 1.14.99.30	ZDS	all-trans-ζ-carotene + an oxidized electron acceptor < = > all-trans neurosporene + a reduced electron acceptor	CV03051B1A05.f1		cassava4.1_004265m	This article
EC 5.5.1.18	LCYe	a carotenoid ψ-end group < = > a carotenoid ε-end group	CV03088A2B02.f1		cassava4.1_005406m	This article
EC 5.5.1.19	LYCb	a carotenoid ψ-end group < = > a carotenoid β-end group	CV03029A1E07.f1		cassava4.1_004296m	This article
EC 5.5.1.19	CasLYCb/partial	all-trans neurosporene < = > β-zeacarotene		ABV01928.1	cassava4.1_006006m	This article
1.14.13.129	BCH1	all-trans-β-carotene + 2 NADH + 2 H+ + 2 oxygen < = > zeaxanthin + 2 NAD+ + 2 H2O	CV03027B1B06.f1		cassava4.1_028637m	This article
EC 1.14.13.129	BCH3	β-cryptoxanthin + NADH + oxygen + H+ < = > zeaxanthin + NAD+ + H2O	CV03027B1B06.f1		cassava4.1_012536m	This article
EC 1.14.13.129	BCH2	all-trans-β-carotene + NADH + oxygen + H+ < = > β-cryptoxanthin + NAD+ + H2O		ABV01927.1	cassava4.1_012554m	This article
EC 1.14.99.45	LUT1	α-carotene + a reduced electron acceptor + oxygen < = > α-cryptoxanthin + an oxidized electron acceptor + H2O	CV03014A2F11.f1		cassava4.1_009189m	Planta 232 (5), 1251–1262 (2010)
EC 1.14.13.90	ZEP1	zeaxanthin + NAD(P)H + H+ + oxygen < = > antheraxanthin + NAD(P)+ + H2O	CV01016B2C04.f1		cassava4.1_015824m	This article
EC 1.10.99.3	VDEP1	antheraxanthin + L-ascorbate + H+ < = > zeaxanthin + L-dehydro-ascorbate + H2O	CV03056B1A12.f1		cassava4.1_034146m	This article
EC 1.14.13.90	ZEP2	antheraxanthin + NAD(P)H + H+ + oxygen < = > violaxanthin + NAD(P)+ + H2O	CV03123B2F01.f1		cassava4.1_003132m	Planta 232 (5), 1251–1262 (2010)
EC 1.10.99.3	VDEP2	violaxanthin + L-ascorbate + H+ < = > antheraxanthin + L-dehydro-ascorbate + H2O	CV03056B1A12.f1		cassava4.1_008263m	This article
EC 1.13.11.51	csZCD	zeaxanthin + 2 oxygen < = > crocetin dialdehyde + 2 (3S)-3-hydroxycyclocitral	CV03025A2B11.f1		cassava4.1_003867m	This article
EC 5.3.99.9	CasNXS	violaxanthin < = > trans-neoxanthin	CV01028B2D03.f1	ABV01925.1	cassava4.1_027255m	This article
EC 1.13.11.51	NCED	9-cis-violaxanthin + oxygen < = > 2-cis,4-trans-xanthoxin + (3S,5R,6S)-5,6-epoxy-3-hydroxy-5,6-dihydro-12′-apo-β-caroten-12′-al	CV03025A2B11.f1		cassava4.1_025279m	This article

Data and information were collected using different public databases including

^a^Refers to Enzyme Commission number (EC number)

^b^Selected possible predicted reaction from PMN http://pmn.plantcyc.org/CASSAVA/NEW-IMAGE?object=CAROTENOID-SYN

^c^Refers to cDNA sequence for microarray elements registered at NCBI http://www.ncbi.nlm.nih.gov/nucest/76606106?report=fasta

^d^Refers to protein sequence registered at NCBI http://www.ncbi.nlm.nih.gov/protein/ACY42665.1

^e^Refers to gene code for protein sequence annotated to cassava proteome data base https://phytozome.jgi.doe.gov/jbrowse/index.html?data=genomes%2FMesculenta&loc=Chromosome08%3A3912601..4149200&tracks=UserBlastResults%2CTranscripts%2CBlatx_Plant_protein&highlight=

^f^Refers to source for information retrieved

#### Carotenoid content variation

A set of 23 landraces representing five different color classes (white, pale yellow, yellow, intense yellow, and pink) was used for direct comparison of carotenoid types identified in CSR extract by HPLC_DAD. Variation in content ranged from 0.4 to 56 μg/g dry weight (DWt) for total carotenoid, and from 0.0 to 33.42 μg/g DWt for total β-carotene (Table 3) across the 21 landraces with variation in yellow color SR. In landrace Cas51 (pink color SR), we detected lycopene as the sole type of carotenoid, at a content of 14.8 μg/g DWt (Table 3). It is important to note the striking variation in the proportion of total β-carotene in relation to total carotenoid across the 21 landraces of cassava with variation in yellow color CSR, which ranges from 21.7 to 76.7 % as showed in Fig. 1. Similar ratios and observations have also been partially reported for CSR of Amazonian landraces before [19].

#### Distribution of carotenoid content in tissue layers of storage root

**Fig. 2:**
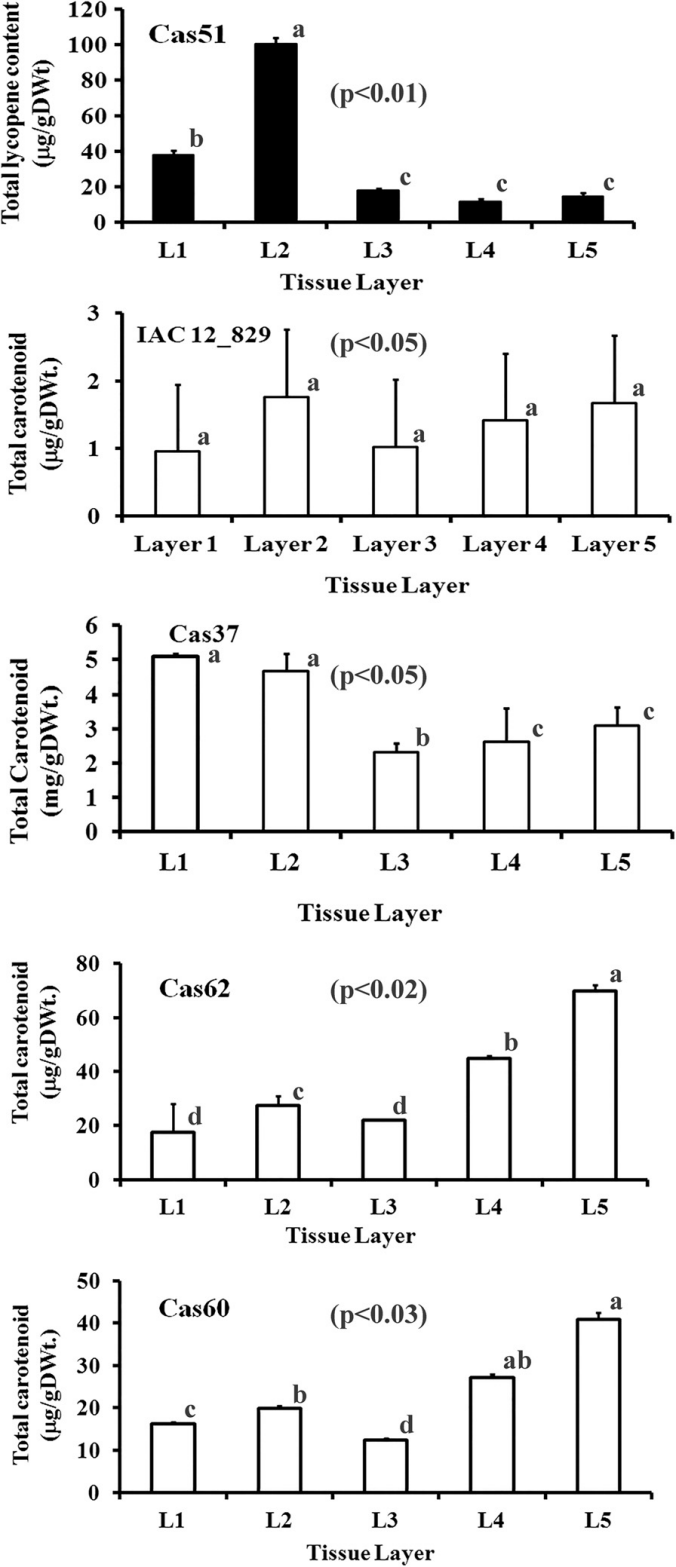
Total carotenoid content distribution across tissue sampling. Tissue layer samples were obtained from a cross section of ten month old storage roots in five landraces with the most contrasting HPLC_DAD profile and variable amounts of total carotenoid. Total carotenoid was estimated using a spectrophotometer for landraces IAC12.829 (White SR), Cas51 (Pink SR), Cas37 (Pale Yellow SR), Cas62 (Intense Yellow SR), and Cas60 (Yellow SR). Values in parenthesis refer to *p*_value (*p* < 0.05) obtained with three biological replications by Kruskal-Wallis statistical test and significance between values is designated by different letters

To gain insight into the nature of developmental mechanisms regulating carotenoid content in CSR, we compared total carotenoid content (Fig. 2), and carotenoid HPLC-DAD profiles (data not shown) for three different tissue samples obtained from the five color classes (P = Cas51, W = IAC12.829, PY = Cas37, Y = Cas60, IY = Cas62) of CSR landraces. Tissue sample I (Layer 1) mainly consisted of phellogen and phelloderm, Tissue sample II (Layer 2) mainly of phloem and cambium, and Tissue sample III (Layer 3, Layer 4, Layer 5) mainly of parenchyma cells and vessels (see Additional file 1: Figure S1). In the central cylinder (Tissue layer III, the edible part of the CSR), total carotenoid in all CSR yellow color groups (Fig. 2) followed the same pattern of accumulation with the lowest amount (μg/g DWt) detected in layer 3 (L3) and increasing amounts in layer four (L4) and layer five (L5). However, the magnitude varied in accordance with the color categories as storage parenchyma cells become older in Tissue sample III, where parenchyma cells in the outer layer 3 are the youngest and those in the innermost layer 5 represent the oldest parenchyma cells. CSR with the most intense yellow color contained the highest amount of total carotenoid in layer 5 (63.59 μg/g DWt), followed in order by yellow (47.60 μg/g DWt, Cas60), pale yellow (2.6 μg/g DWt, Cas37), and white CSR (2.30 μg/g DWt, IAC12). Carotenoid HPLC-DAD profiles (not shown) as well as proportions of total β-carotene in different Tissue samples were equivalent and correspondent to those observed in bulk preparations of CSR. An exception was the case of lycopene distribution in landrace Cas51, which contains an increased amount of lycopene (100.31 μg/g DWt) in layer 2 (Fig. 2) and equal amounts in each of the three layers in Tissue sample III.

This observed pattern of total carotenoid accumulation in different cell zones (L3, L4, L5) of tissue sample III is the first report showing corresponding values when total carotenoid content was estimated in bulk storage root of these landraces. Data presented in Fig. 2, resulting due to tissue age, suggests that changes in carotenoid profiles and total content is quantitative rather than a change in the amount of a single carotenoid type. Collectively, this spatial pattern of carotenoid accumulation reveals a close association of tissue age in the central cylinder (Tissue sample III) of CSR and color intensity variation as secondary growth proceeds.

### Cloning, sequencing and identification of genes coding for enzymes in the carotenoid synthesis pathway

**Fig. 3:**
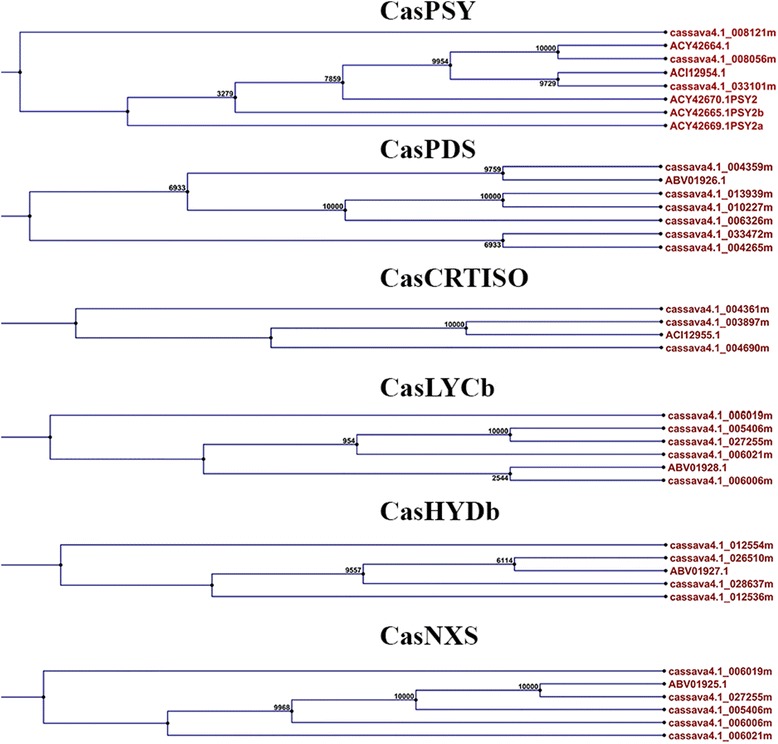
Phylogenetic analysis of six protein sequences deduced from cDNA belonging to the carotenoid biosynthesis pathway from cassava storage root. Sequence names on the tree branches refer to protein codes in NCBI (http://www.ncbi.nlm.nih.gov/nucest/7660610) and cassava proteome assignment (https://phytozome.jgi.doe.gov/pz/portal.html). Symbols refer to common enzyme name

cDNA fragments of genes coding for enzymes PSY, PDS, CRTISO, BCH, LYCb, and NXS were cloned from CSR extracts, sequenced and registered in GenBank [26]. These genes were unevenly distributed in the cassava genome database [27], based on the variable number of matching cDNA fragments. Therefore, protein sequences were aligned and a neighbor-joining phylogeny tree was generated to confirm the association of their sequence with specific enzymes described in other plants (Fig. 3). The results indicated that CasPSY sequences matched with eight protein-coding loci of the cassava genome with three sequences annotated as PSY2 in the NCBI database [26]. Predicted protein fragments for CasPDS (ABV01926.1), CasCRTISO (ACI12955.1), CasLYCb (ABV01928.1), CasHYDb (ABV01927.1), and CasNXS (ABV01925.1) matched with 6, 3, 5, 4, and 5 loci of the cassava genome database [27], respectively. Furthermore, each one of the genes presented have complete identity with a primary locus in the cassava genome [27], including cassava4.1_003897 for *CasCRTISO*, cassava4.1_004359 for *CasPDS*, cassava4.1_012554 and cassava4.1_012536 for *CasBCH(=CasHYDb)*, cassava4.1_006021, cassava4.1_005406, cassava4.1_006019, cassava4.1_027255, and cassava4.1_006006 for *CasLYCb*, as well as cassava4.1_027255 for *CasNXS*. These results confirm the identity of each cDNA fragment obtained in this work. Finally their sequences were used for primer design and subsequently used for qRT_PCR expression analysis.

### Gene expression analysis in landraces of most contrasting HPLC-DAD profiles

#### Transcript abundance associated with carotenoid synthesis and cleavage investigated by microarray

**Fig. 4:**
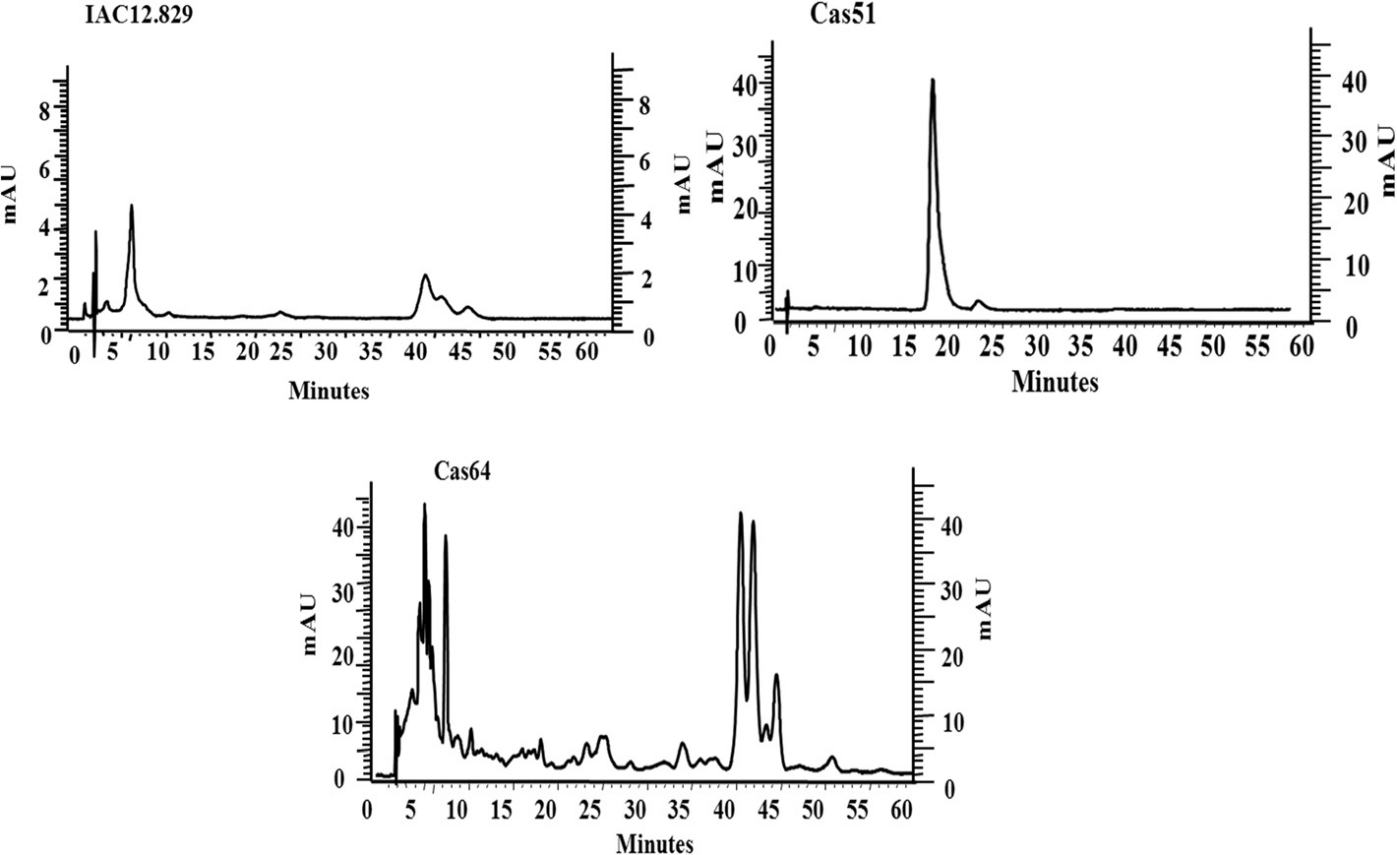
Most contrasting HPLC_DAD carotenoids profiles. Carotenoid profiles for 3 of the 4 most contrasting HPLC_DAD carotenoid profiles from a total of 23 landraces studied. Carotenoid profiles were obtained by HPLC_DAD using a C18 Waters Spherisorb ODS_2 (4.6 × 150 mm, 5 mm) column and mobile phase of Methanol: Ethyl Acetate: Acetonitrile (1:1:8) with a flow rate of 1 mL per minute during 60 min in an isocratic run. Carotenoid types were identified using reference profiles (Additional file 3: Figure S2) and standard profiles (Additional file 4: Figure S3)

Genes coding for enzymes of the carotenoid synthesis and cleavage pathways in cassava have been reported at the Plant Metabolic Network [31]. Here, microarray analysis was used to determine transcript abundance of annotated genes in the cassava genome for three landraces showing the most contrasting carotenoid HPLC-DAD profiles (Fig. 4).

**Fig. 5:**
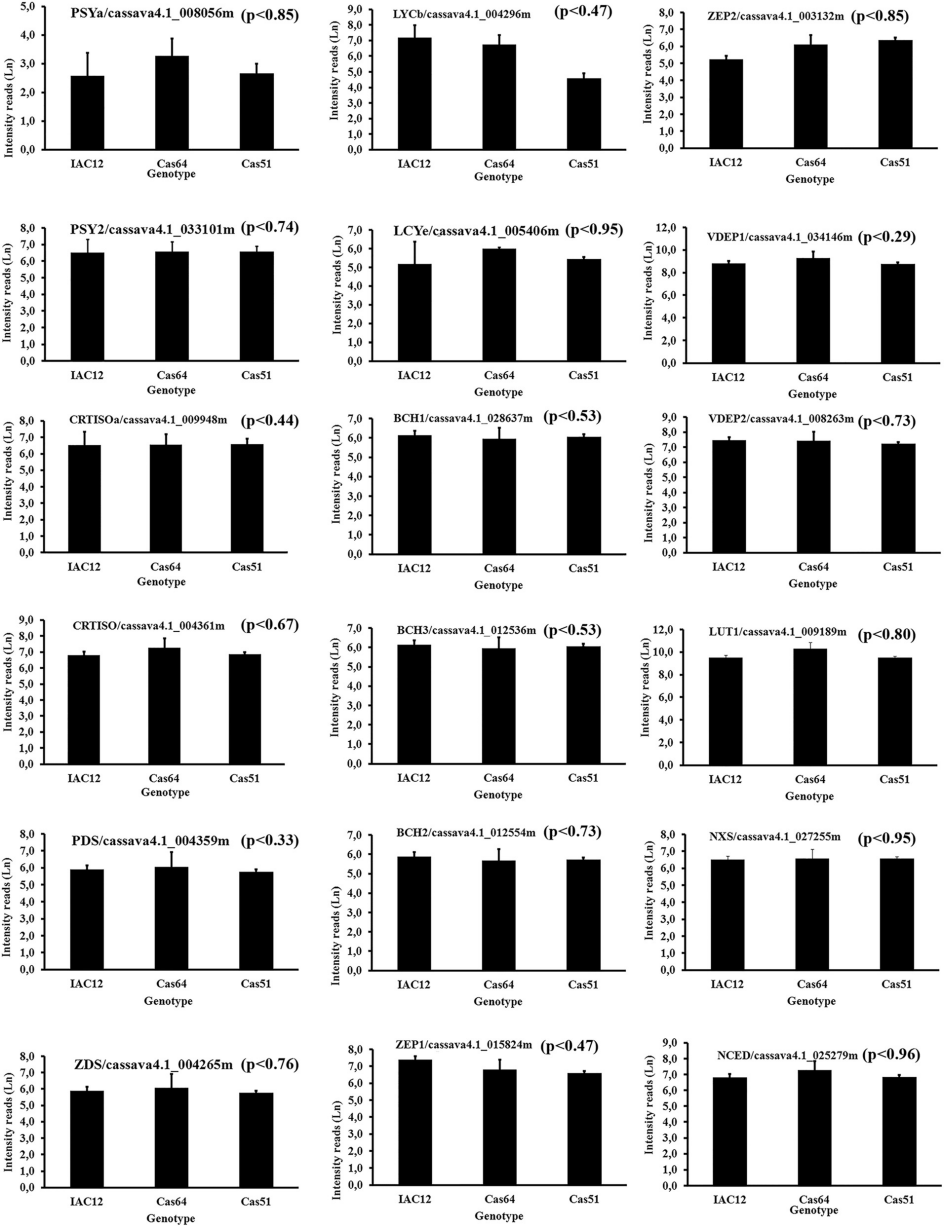
Transcript abundance for 18 genes coding for enzymes/proteins related to carotenoid biosynthesis in cassava storage root. Transcript abundance based on microarray analysis. Three landraces representing three distinct color classes (W-IAC12, IY-Cas64, P-Cas51) showing the most contrasting HPLC_DAD carotenoid profiles as shown in Fig. 4. Values refer to natural log for microarray fluorescence readings. Enzyme abbreviation and gene code in cassava genome are shown. Values in parenthesis refer to p_value obtained with three biological replications by Kruskal-Wallis statistical test. No significance between values were detected

**Fig. 6:**
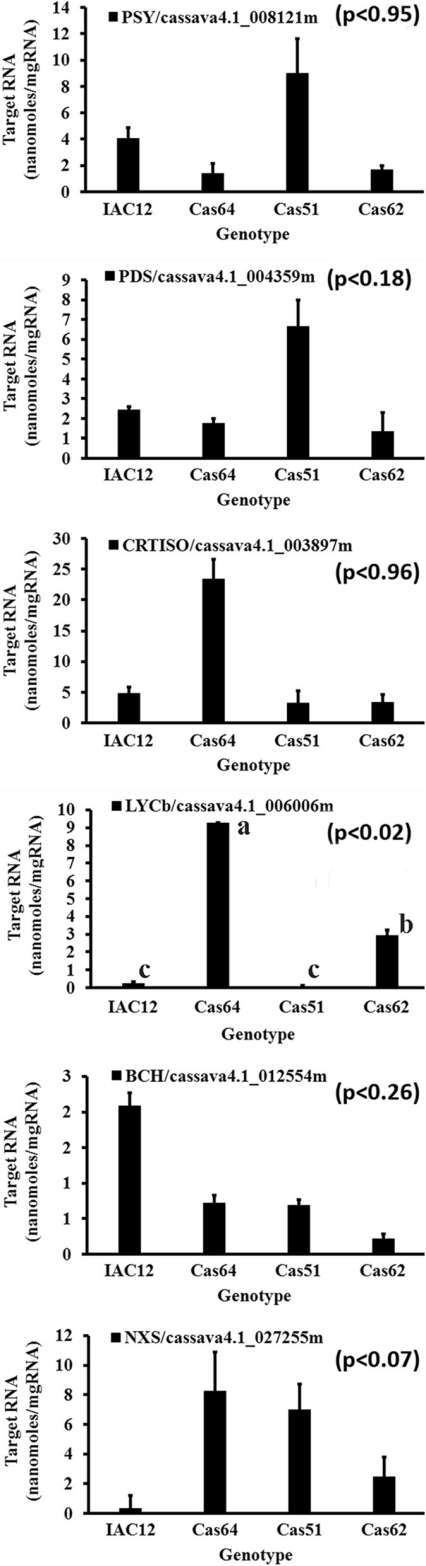
Transcript abundance for 6 major genes coding for enzymes/proteins related to carotenoid biosynthesis in cassava storage root. Transcript abundance based on qRT_PCR analysis. Four cassava landraces are representing four color classes (W-IAC12, IY-Cas64 and Cas62, P-Cas51) with the most contrasting HPLC_DAD carotenoid profiles. Values refer to measured cDNA amplified amount of target RNA (nanogram/mgTotal RNA). . Values refer to natural log for microarray fluorescence readings. Enzyme abbreviation and gene code in cassava genome are shown. Values in parenthesis refer to p_value (*p* < 0.05) obtained with three biological replications by Kruskal-Wallis statistical test and significance between values is designated by different letters

**Fig. 7:**
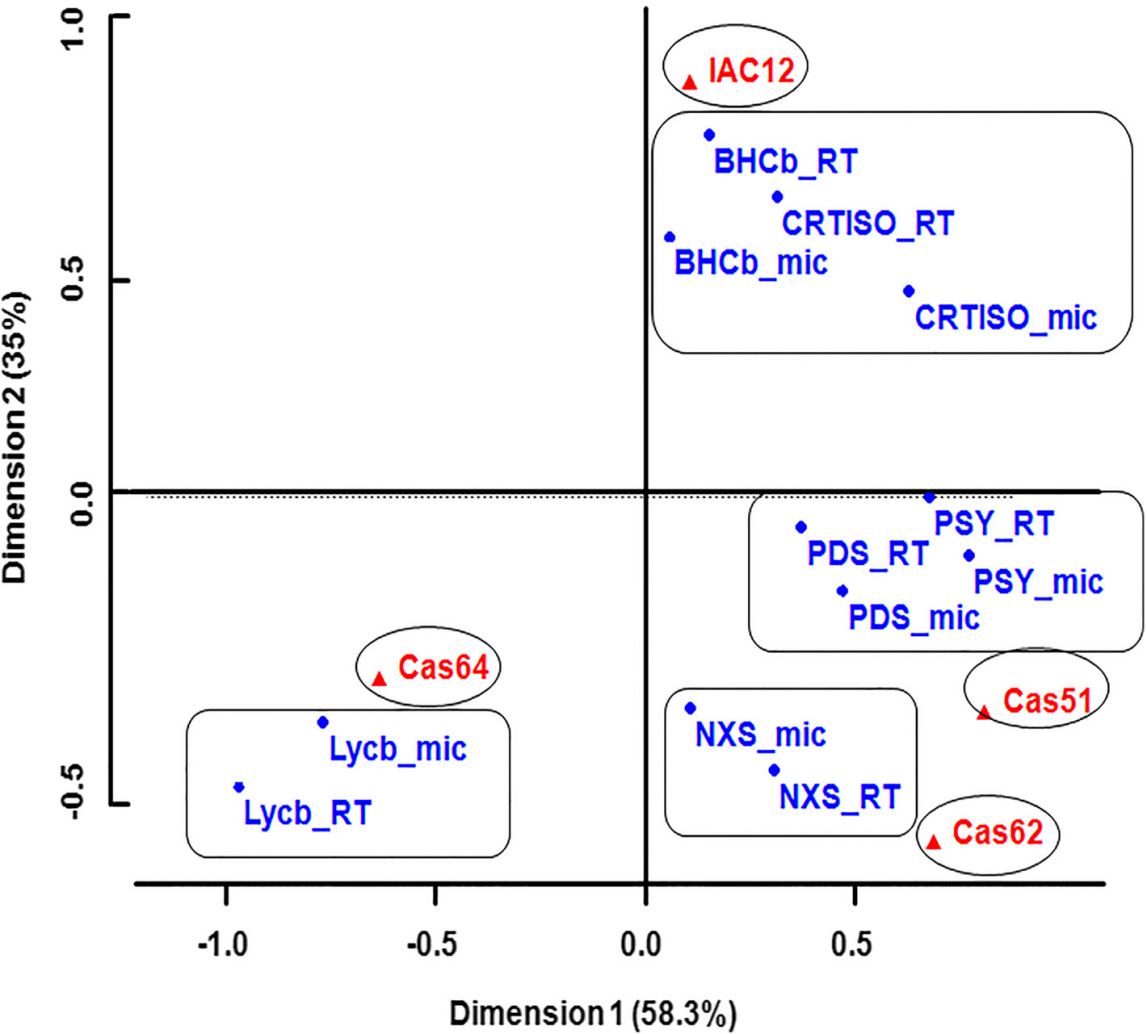
Association of transcript abundance measured by microarray to that by qRT-PCR for six genes in landraces with contrasting HPLC_DAD profiles. Graph shows the experimental data and the best-fit correspondence values for microarray and qRT_PCR gene expression measurements for four landraces (IAC12, Cas64, Cas62, and Cas51) with contrasting total carotene accumulation with three biological replications. Note the range of results with excellent agreement between microarray data and qRT-PCR. Abbreviation for enzyme names are accompanied with the symbol for the microarray (mic) and qRT_PCR (RT). Detailed statistical data treatments are described in Methods. Values refer to natural log (Ln) for microarray fluorescence readings. The correspondent gene code in cassava genome for the enzymes abbreviation is as in Table 5

Cassava storage root transcript profiles (Fig. 5) were accomplished by probing a cDNA microarray, containing 25,395 entries as previously described [28–30] with labeled fragments from total RNA extracts of landraces IAC12, Cas64 and Cas51, which had the most contrasting HPLC_DAD profiles. Similarly, qRT_PCR was performed using primers specific to cDNA coding for the six major enzymes in the predicted carotenoid biosynthesis pathway (Fig. 6). This pattern is consistent with the content of β-carotene and lycopene in both Cas64 and Cas51 landraces; as well as IAC12 and Cas62, as revealed by correlation studies with transcript abundance obtained by qRT-PCR (Table 4) and microarray data (Table 5). In addition, the correspondence analysis for measurements of mRNA by microarray to that by reverse transcription PCR (RT-PCR) strengthen the association between the level of specific gene expression and a particular landraces genetic background. First the total variation is 93 % explained for this association, being 35 % by dimention1 and 58.3 % by dimention2 as observed in Fig. 7, which forms four distinct groups of correspondence that are significantly (χ^2^-value = 109.1492, *p*-value = 4.443e^−10^) associated with a particular landrace (Table 6). Therefore, gene expression measurements are of equivalent values and trend of association (Fig. 7) with genetic background diversity revealed by SR color and contrasting HPLC-DAD chromatograms profiles for the four landraces studied.

**Table 4:** Correlation parameters revealed among different classes of carotenoid types and abundance of transcripts coding for six major enzymes based on qRT_PCR

Total acyclic carotenoid correlation			
Enzyme	cDNA code in microarray data base	Cassava genome code	*p* value
ZDS	CV03051B1A05.f1	cassava4.1_004265m	0.109
Total β-carotene correlation			
LCYe	CV03088A2B02.f1	cassava4.1_005406m	0.067
NCED3	CV03105A1F08.f1	cassava4.1_029535m	0.046
PDS	CV03049A2F07.f1	cassava4.1_004359m	0.037
PDS	CV03110A2H07.f1		0.139
ZEP	CV03090B2B05.f1		0.003
ZEP	CV03104A2A10.f1		0.009
Total Xanthophyll correlation			
LCYe	CV03127A2F10.f1		0.007

Carotenoid intermediates were pooled based on classes of carotenoid end products including cyclic (β-carotene + β-xanthophylls), and acyclic (phytoene + phytofluene + ζ-carotene + neurosporene + β-zeacarotene + lycopene) carotenoids. Pearson product moment correlation coefficient (**r**) was obtained for linear association of total cyclic, total acyclic, total β − carotene and total xanthophylls against abundance of transcripts coding for enzymes related to carotenoids synthesis. The values of **SE of** **r** accounts for the standard error of the estimates by the linear regression and represents the level of accuracy of predictions. If probability that *r* = 0, P(*r* = 0) is < =0.05, r is significantly different from 0 and some degree of correlation is shown. The value **n** refers to number of replication

**Table 5:** Most significant p-values revealed by the association between classes of carotenoid types and abundance of transcript coding for enzymes related to carotenoid biosynthesis obtained with microarray data

Enzyme	Correlation (r)	S.E. of r	Significance *P* (*r* = 0)	*n*
Total Cyclic Carotenoids				
PSY	0.262	0.042	*	5
PDS	0.513	0.438	ns	5
ZCD	0.420	0.200	ns	5
CRTISO	0.486	0.347	ns	5
LYCb	0.073	0.001	***	5
HYb	0.575	0.885	ns	5
NXS	0.575	0.877	ns	5
TOTAL Acyclic Carotenoids				
PSY	0.648	0.597	ns	5
PDS	0.611	0.496	ns	5
ZCD	0.706	0.954	ns	5
CRTISO	0.424	0.200	ns	5
LYCb	0.703	0.893	ns	5
HYb	0.616	0.509	ns	5
NXS	0.616	0.508	ns	5
TOTAL β − carotene				
PSY	0.423	0.199	ns	5
PDS	0.664	0.655	ns	5
ZCD	0.536	0.348	ns	5
CRTISO	0.625	0.531	ns	5
LYCb	0.048	0.002	**	5
HYb	0.707	0.960	ns	5
NXS	0.706	0.952	ns	5
TOTAL Xanthophylls				
PSY	0.343	0.126	ns	5
PDS	0.052	0.003	**	5
X ZCD	0.653	0.616	ns	5
CRTISO	0.701	0.870	ns	5
LYCb	0.663	0.653	ns	5
HYb	0.264	0.072	ns	5
NXS	0.259	0.0706	ns	5

Carotenoid intermediates were pooled based on classes of carotenoid for end products of group of reactions type as illustrated in Fig. 2. Pearson product moment correlation coefficient (**r**) was obtained for linear association of total cyclic, total acyclic, total β − carotene, and total β_xanthophylls content (μg/g DWt) against abundance of transcripts coding for enzyme related to carotenoids synthesis. The values of **SE of** **r** accounts for the standard error of the estimates by the linear regression and represents the level of accuracy of predictions. If probability that *r* = 0 P(*r* = 0) is < =0.05, r is significantly different from 0 and some degree of correlation is shown. *significant, **highly significant, ***most significant. Relevant cDNA sequences were annotated to the cassava

**Table 6:** Statistical parameters for the correspondence analyses of transcript abundance evaluated by microarray in relation to that by qRT_PCR

χ^2^_values	df	*p*-value
109.1492	33	4.443e^−10^
Dim1: 35 % , Dim2 = 58 %		

Levels of significance for χ^2^_values and p_values for the correspondence of measurements of transcript abundance either by microarray or qRT_PCR assay. Transcripts coding for the six major enzymes (PSY, BHCb, CRTISO, PDS, LYCb and NXS) in the carotenoid synthesis pathway across four landraces (IAC12, Cas62, Cas64, and Cas51) showing a range of total carotene accumulation.

#### Association between transcript abundance and plastid multiplication investigated by microarray

**Fig. 8:**
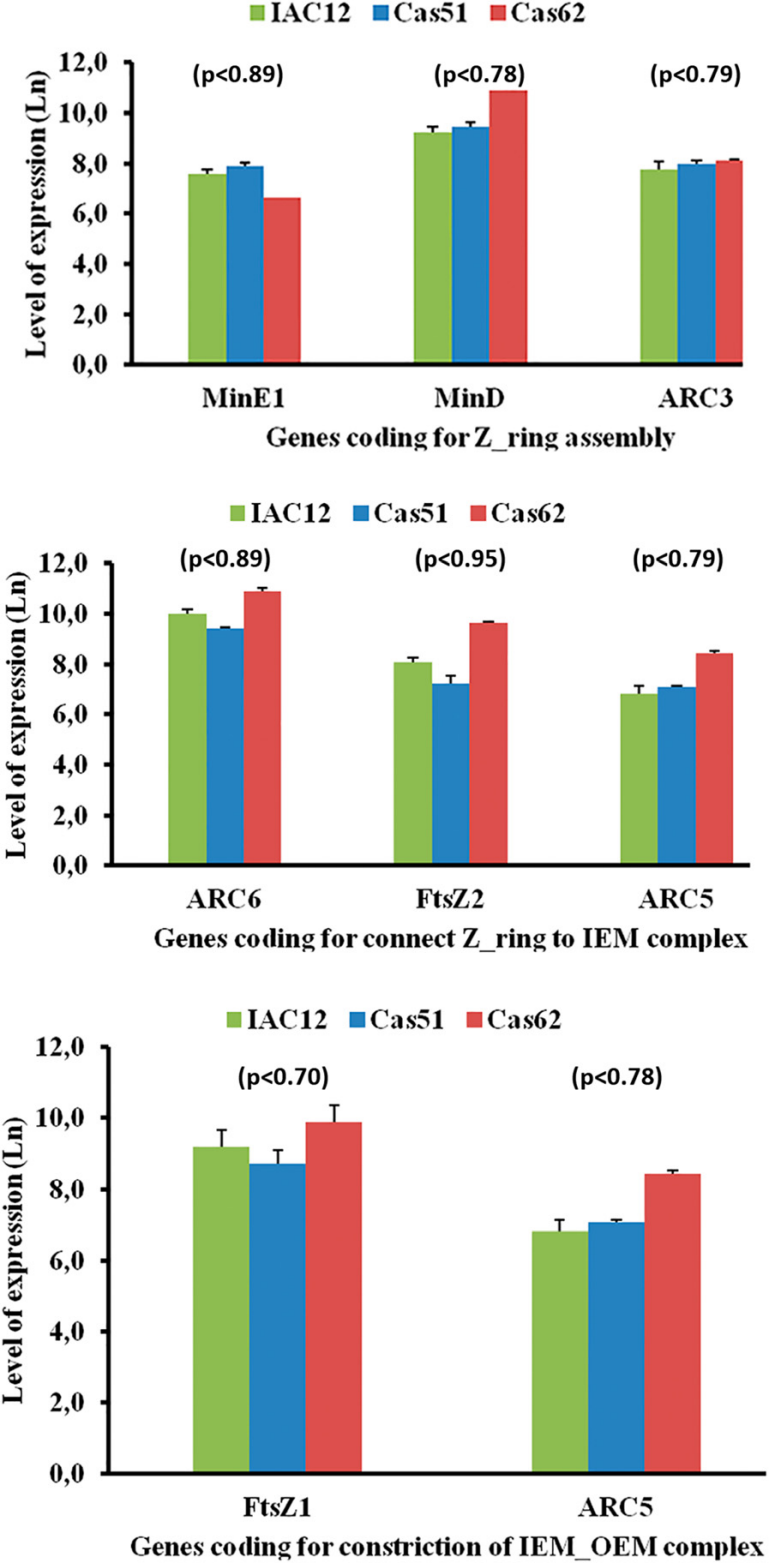
Abundance for seven major transcripts coding for proteins related to plastid multiplication. Three cassava landraces representing three color class (W-IAC12, IY-Cas62, P-Cas51) with the most contrasting HPLC_DAD carotenoid profiles and β-carotene accumulation. Protein name abbreviation (MinE1, MinD, ARC3, ARC6, ARC5, FtsZ1 and FstZ2) and their respective gene code (cassava4.1_015317m, cassava4.1_011379m, cassava4.1_001913m, cassava4.1_028805m, cassava4.1_002180m, cassava4.1_006542m, and cassava4.1_006542m) annotated in the cassava genome. Values refer to natural log for microarray fluorescence readings. Values in parenthesis refer to *p*_value (*p* < 0.05) obtained with three biological replications by Kruskal-Wallis statistical test. No significance between values were detected

To determine possible correlations between differential accumulation of specific carotenoid types and chromoplast abundance, transcripts coding for proteins related to plastid multiplication were accessed through our microarray data set. Based on a model for plastid division [37–39] that involves 13 proteins involving complex interactions during three steps of plastid multiplication, we investigated the transcript abundance for eight of these proteins using three landraces with the most contrast in β-carotene accumulation. Three genes coding for proteins MinE1, MinD, ARC3 (account for the assembly of the Z-ring formation), three genes coding for proteins ARC6, FtsZ2, ARC5 (account for the attachment of the Z-ring to the inner envelope membrane), and two genes coding for proteins FtsZ1, ARC5 (account for the constriction of the outer envelope membrane). Transcripts abundance (Fig. 8) indicated overall equivalent values across the tested landraces, which is consistent with their coordinated action in each step regarding the plastid multiplication in landraces with contrasting β-carotene accumulation. However, some differential transcript abundance was observed for the genes coding for MinD, ARC6, FtsZ2, ARC5, and FtsZ1 among landraces Cas51, IAC12, and Cas62, which had the most contrasting HPLC_DAD carotenoid profiles and accumulated different amounts of lycopene and β-carotene, respectively.

## Discussion

### Identification of carotenoids from cassava storage root

Analytical methods exploiting photo-diode array detection (DAD) use UV/VIS spectra to elucidate structural characteristics of carotenoids, which aids in the identification of specific carotenoid types [40, 41]. DAD technology in combination with HPLC carotenoid separation on a C18 column [40, 41] allowed us to separate and detected 27 peaks; however, in our results, not all the carotenoid peaks could be identified. Further research is therefore recommended in order to confirm the identification of all 27 peaks observed in CSR.

### Carotenoid diversity

To our knowledge, the data presented here provide the first report reflecting the dynamics of the carotenoid synthesis pathway in storage root across cassava landraces. The results partially account for the differential abundance of 19 carotenoids associated with either yellow-color SR, or of lycopene, the sole carotenoid detected in pink CSR (Cas51). This work also revealed variations in (1) the proportion of particular carotene types in relation to total carotenoids, (2) the presence of variable carotenoid intermediates across the landraces studied, and (3) their association with abundance of transcripts coding for key enzymes in the carotenoid synthesis and cleavage pathway in landraces (IAC12, Cas64 and Cas51) showing the most contrasting HPLC_DAD profiles. Thus, the variation in CSR color in the studied landraces, ranging from white (IAC12) to intense yellow (Cas64) and pink (Cas51), could be directly related to the presence of different carotenoid types.

The cassava landraces used in this study primarily accumulated β-carotene, albeit in vastly different amounts and proportions in relation to the intensity of the yellow color CSR. One exception occurs for the SR of landrace Cas51 (pink color CSR) that accumulates only lycopene, which indicates that individual steps of the carotenoid pathway may be blocked. In the case of color variation resulting from an increased content of β-carotene in landrace Cas62 (the most intense yellow SR), the effect is primarily quantitative and implies that individual steps in the carotenoid biosynthetic pathway are not blocked and that downstream accumulation of intermediates does not occur. Another factor that may contribute to the gradient in intensity of yellow CSR across landraces is the variation in ratios of α- and β-ring xanthophylls and total β-carotene. Most of the detected xanthophylls are of β-ring type with low amounts of lutein, which is the end product of the α-ring branching pathway. With regard to this variation, landrace Cas64 contained mainly β-carotene and traces of β-cryptoxanthin, landrace Cas37 contained mainly β-carotene and lutein, and landrace Cas33 mainly β-carotene and β-ring xanthophylls. Although landraces with white CSR have no visible yellow color, it has an equivalent proportion of β-carotene, albeit with extremely low levels of total carotenoid and β-carotene (Table 3).

In contrast to white and yellow color CSR, a landrace with pink color CSR (Cas51) appears to result from the presence of high lycopene content. Some of these carotenoids have already been identified in CSR, including β-carotene and its isomers [23, 24], phytoene, phytofluene, xanthophylls [24], and lutein [42, 43]. However, to the best of our knowledge zeaxanthin, antheroxanthin, violaxanthin, neoxanthin and crocetin presence in CSR are first reported here.

**Fig. 9:**
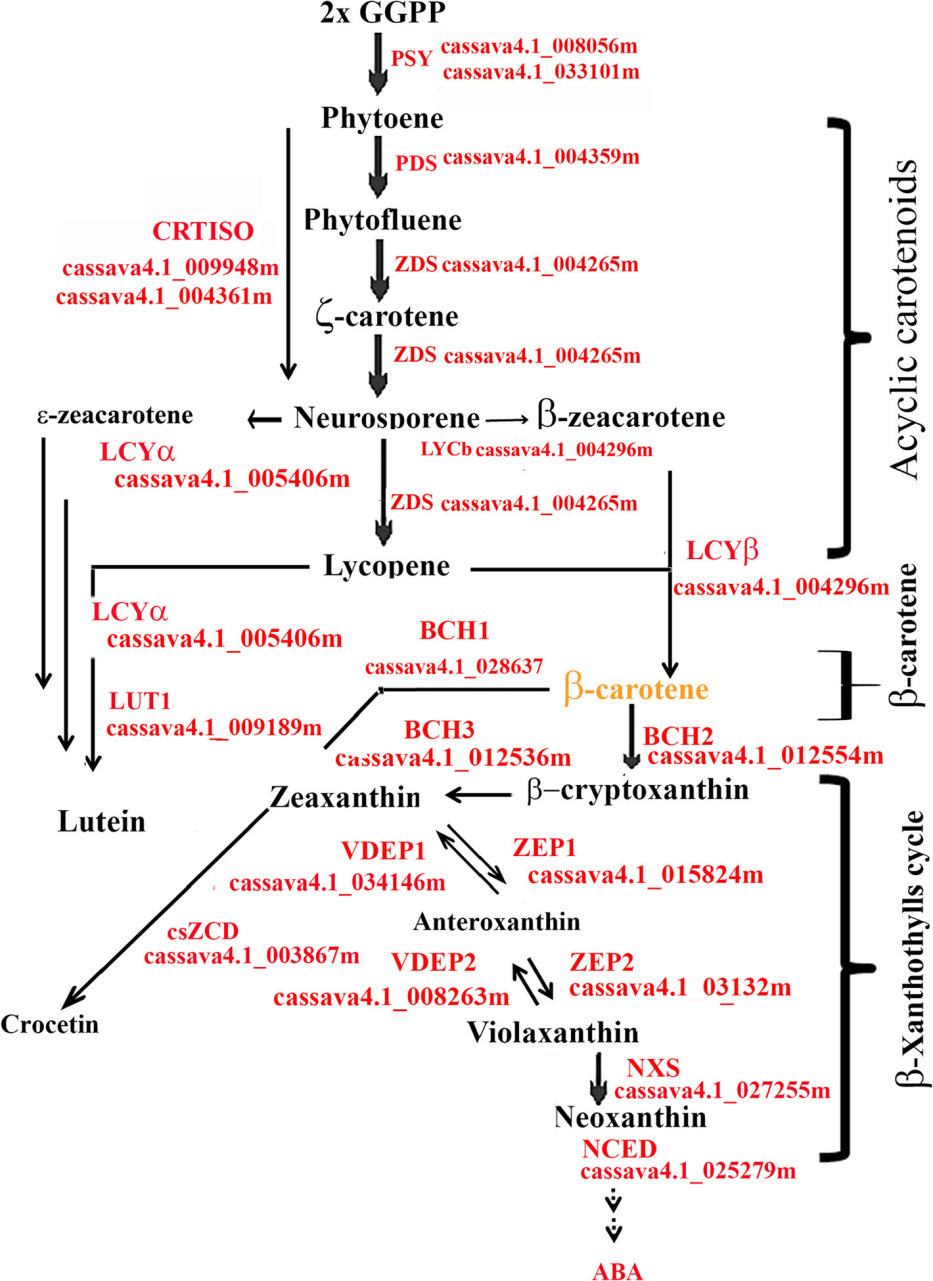
Proposed diagram for carotenoid biosynthesis pathway in cassava storage root based on qRT_PCR gene expression analysis. Carotenoid biosynthesis pathway was designed based on carotenoid intermediates identified by isocratic run using a C18 column separation with HPLC_DAD identification system, abundance of transcripts coding for proteins/enzymes, and their code identification in the cassava genome. Analytical and diagram assembly were performed as described in Methods

Based on the information retrieved from the carotenoid biosynthesis super pathway reported at PNM site [31] and shown in Table 1, the carotenoids types identified above, and 23 expressed genes coding for carotenoid synthesis enzymes annotated in the cassava genome [27], we designed a carotenoid biosynthesis pathway diagram (Fig. 9). This suggests that differences in predicted expression patterns of carotenoid synthetic pathway genes are related primarily to the regulation of pathway flux through downstream intermediates in the yellow color CSR groups and not due to blocking specific steps in the pathway, as appears to be the case in the pink CSR of Cas51. Variation in the types of carotenoids includes differential presence of total cyclic, acyclic, α-ring and β-ring carotenoids, which account for variation in the branching of the carotenoid biosynthetic pathway and xanthophylls cycle. For instance, β-carotene is the major carotenoid accumulated in yellow CSR, but was present in variable proportions in relation to total carotenoids, ranging from 31.8 to 76.78 %. This is distinct from other non-green organs systems in plants such as potato tuber [44] that accumulates mainly zeaxanthin (51 %), antheraxanthin (25 %), and violaxanthin (11 %), and carrot that accumulates mainly α- and β-carotene [45]. The carotenoid composition closest to that in CSR is found in sweet potato SR, which accumulates mainly β-carotene, like cassava, but differs in the pattern of the geometric stereoisomers of β-carotene. While cassava contains 9-*Z* and 13-*Z* (50 %), and all-*E* (50 %) as natural isomers, sweet potato contains 9-*Z* isomers [46] in a different proportion in relation to their equivalent counterpart. Yet, the differences in yellow color intensity are largely due to the presence of different carotenoid types, other than β-carotene, rather than due to the accumulation of different amounts of a specific carotenoid type. Therefore, non-photosynthetic tissues, including, potato tuber [44], carrot [45], sweet potato [46], flowers [47], and watermelon [48] show divergent patterns in carotenoid content, in comparison with CSR.

Overall, the carotenoid synthesis pathway is fully active in all CSR color types (Table 3), but with extremely low accumulation of total carotenoid in white CSR. Possible explanations for this include protein-protein interaction within unknown intrinsic enzyme activities, *in vivo* accumulation of substrate for a particular enzymatic step in the pathway, lack of a direct relation between gene expression and intrinsic enzyme activities, as well as SNP variation in a specific gene sequence as demonstrated for PSY2 [42, 43]. Although we have not ruled out any of these possibilities, this variation may be related to physiological requirements of some downstream derivatives of the apocarotenoid pathway that may contribute to increased turnover within this pathway. Another example includes the apocarotenoid derived from either violaxanthin, neoxanthin, or ABA, which is a well-known hormone involved in environmental stress responses and signaling in plants [49], including cassava [43]. Considerable amounts of violaxanthin and neoxanthin were observed in CSR of landraces adapted to wet environments in the Amazon that exhibit varying intensities of yellow CSR. However, this is not the case for white cassava CSR landraces, which are widely cultivated across the seasonal cold and dry environment of Cerrados. Alternatively, the white color may be related to the low capacity of white CSR to accumulate β-carotene via protein sequestration in chromoplasts, as observed in other plant systems [50] and recently reported for cassava storage root [51]. Indeed, this observation corroborates with the suggestion that HSP21 plays an important role in the increased accumulation of β-carotene in landrace Cas62 [51].

The α-carotene, a precursor of lutein, was not detected in the group of landraces with yellow CSR in this study. However, lutein was detected in most of the yellow color landraces except landrace Cas62. This might also be the result of an alternative pathway or interconversion of lutein and xanthophylls, as speculated [52]. The amount of metabolic intermediates such as phytofluene varied from zero in the majority of the landraces studied to 2.3 μg/mg DWt in Cas56 and 15.7 μg/mg DWt in Cas62. Results observed in qRT_PCR indicates, that this variation could be related to intrinsic enzyme activity and interaction of CasPSY, CasCRTISO, and CasPDS, as indicated by the significant level of correlation between their transcript abundance and the carotenoid content reported here and speculated elsewhere [14]. Collectively, these findings provide important information on the genetic background of specific germplasm from the Amazon (center of origin and domestication of cassava), with regard to improving the β-carotene content in commercial cassava, either by conventional breeding [14, 16] or by demonstrated transgenic approaches [42, 43].

### Spatial carotenoid accumulation in storage root

Based on cassava storage root anatomy, some spatial age-regulated processes have been proposed [53–55] and validated in CSR. These include apparent amylose content [56], free sugar and starch accumulation in *sugary* cassava [57], and expression levels of *Mec1*, which is known to be involved in secondary xylem maturation [25]. Tissue sample III (i.e., the central cylinder of the storage root), the edible part of CSR, is of particular interest because it comprises the major storage parenchyma cells for accumulation of starch and carotenoids. Because tissue sample III originates from cambium activity as the adventitious root swells [55], it is possible to identify storage parenchyma cells of different ages, as they progressively arise from the outer side of the cambial meristem and age toward the inner diameter. Therefore, parenchyma cell layers in tissue sample III were hand dissected, divided in cell zones and referred to as L3 (young parenchyma), L4 (intermediate age parenchyma) and L5 (old parenchyma). Here, for the first time, we describe a spatial pattern of carotenoid accumulation, corresponding to a temporal pattern, in that it is closely associated with secondary parenchyma cell age in the central cylinder (i.e. the cell zones in tissue sample III) and with color intensity variation in CSR cassava landraces. The variable pattern observed may be due to three concomitant events. First, carotenoid accumulation is dependent on parenchyma cell age during secondary growth of CSR. This means that carotenoid content variation is independent of dry matter accumulation and organ age. Second, carotenoid accumulation proceeds at a lower rate than the increase in dry matter. Third, the similarity of carotenoid profiles among cell zones of tissue sample III in all color categories and the dependency of carotenoid accumulation on parenchyma cell age indicate that carotenoids are first synthesized and later stored in CSR.

### Genetic analysis based on pattern association of gene expression

The limited amount of information on the genetic control of carotenoid biosynthesis in cassava underscores the need for further studies to explain the observed diversity in the present report. In the hypothesis that two genes with epistatic effects control the yellow color of CSR [58] it is assumed that the *Y1* gene is responsible for the transport of carotenoid into the storage root. However, results from the present study suggest that this control is much more complex, and that *Y1* may not be of *in vivo* relevance, due to the large diversity detected and the distribution of carotenoid content across storage root tissue layers. It is plausible that several other genes are involved in the synthesis, as has been observed in carrot [45], in which ten out of twenty one genes corresponding to the color phenotype are related to the synthesis of carotenes [15], and their requisites for protein [50] as well as lipid [50] accumulation.

Here, we provide genetic evidence based on pattern association of transcript levels for six major genes, either by qRT_PCR or microarray results as visually detected in Fig. 9 and actual values presented in Figs. 5 and 6 for four landraces showing a range of variable total carotenoid content. It is observed that the level of gene expression is highly dependent on the natural genetic background variation of the landraces. For instance, landraces Cas64 and Cas62, with intense yellow SR, have correspondent expression for *LYCb* and *NXS* respectively, while landraces with white (IAC12) and pink (Cas51) colored SR have correspondent measurements for *BCHb* and *CRTISO* (IAC12) and *PDS* and *PSY* (Cas51) respectively, and undetected values for *LYCb* in both genotypes. Therefore, it is striking that the associations are coherent with the chromatographic profiles among these four landraces showing contrasting carotenoid content and type in their SR. While the measurements of *LYCb*, either by microarray or qRT_PCR, are correspondent for intense yellow SR landrace Cas64, which has 16 peaks in the HPLC_DAD carotenoid chromatogram, it is absent in pink SR of landrace Cas51 with its single peak identified as lycopene. Similarly, the low values of *NXS* for intense yellow SR of landrace Cas62, which accumulates the highest amount of β-carotene, suggests that down regulation of the carotenoid pathway through decreased expression of *CasBCH* in SR of this landrace probably affects the expression of *NXS* downstream in the pathway.

Other variations related to the accumulation of specific carotenoids could not be explained in a similar way. Because plastids are the site for both synthesis and accumulation of carotenoids, it is likely that chromoplast number and size would also change to accommodate decreased or increased amounts of carotenoids, as observed in this study. Here we assessed this possibility by exploiting a comparative microarray analysis based on abundance of transcripts coding for proteins regulating plastid replication using three landraces with most contrasting levels of β-carotene. As depicted in Fig. 8, abundance of transcripts corresponding to proteins involved in all three steps of plastid replication are increased in landrace Cas62. Exceptions include abundance of *MinE1* and *ARC3*, coding for proteins involved in the Z-ring assembly, which were not increased. The increased abundance of *MinD* (involved in Z-ring assembly), *ARC6* (involved in connecting the Z-ring to the IEM complex), and *FtsZ1* (involved in constriction of IEM_OEM complexes) suggest critical roles for their corresponding proteins in plastid replication processes in CSR. This might help explain the increased levels of β-carotene in landrace Cas62 CSR. This may also explain the variation in levels of β-carotene across the 21 yellow CSR landraces studied, which might be due to the number of chromoplasts [39]. Further research is underway to identify the nature of the genetic diversity in the two phenotypes observed in Cas51 and Cas62, using a combination of crossings between these landraces and commercial white cassava.

The nutritional value arising from the carotenoid diversity observed in this study is of three fold significance. First, β-carotene is the major carotenoid present (31.8–76.7 %) across the landraces studied. The identification of Cas62 as a landrace with a high β-carotene content may rank cassava as an important single source of retinol equivalents in a staple food crop. Second, the presence of lutein in Cas56 may designate this landrace as a significantly valuable diet for protection against the onset of age-related macular degradation. Third, Cas51 might represent a valuable source of lycopene, which is accumulated in high amounts in this landrace. Collectively, the results of this study demonstrate the natural variance in abundance of transcripts coding for specific key enzymes by qRT_PCR in the carotenoid biosynthesis pathway, as well as divergence in carotenoid metabolic flux toward distinct products in different landraces of cassava. In addition, processes associated with regulation of plastid biogenesis, observed in this study, could help with correlations to carotenoid accumulation in CSR. However, further studies on the impact of chromoplast number and size on specific carotenoid accumulation in CSR are needed.

## Conclusions

Genetic variation in the color of CSR from the Amazon, the center of origin and domestication of cassava, offers an important resource for the investigation of the carotenoid biosynthesis pathway in its native state. We further demonstrate that HPLC-DAD profiling is suitable for the identification of the major carotenoids important for improvement of cassava nutritional values, aided by the existing natural diversity. Although we could not evaluate intrinsic enzyme activities, the level of transcript abundance in association with extreme variations in carotenoid types and contents allowed us to propose a carotenoid biosynthesis pathway for CSR based on the data presented here. Finally, although gene expression alone limits determination of the genetic nature of these data, information can be gained by performing crossbreeding involving the landraces with these reported phenotypes to establish potential mutations in *LCYb* for the pink color landrace Cas51, and *BCH2* for the IY color landrace Cas62. Indeed, this approach is currently underway and will yield important data that may enable us to determine the type of natural mutations occurred in these two landraces. Information related to variation of total carotenoid content due to tissue age will also contribute to improve the accuracy in sampling tissues for genetic analysis. Results presented here are a first step toward this goal, which now can be achieved using tools that assist in identifying spontaneous mutations impacting carotenoid presence and function in a non-green tissue of a stable root crop like cassava.

## Abbreviations

ABA, abscisic acid; ARC3, is a protein encoded by the ARC3 gene in plastid division; ARC5, is a protein encoded by the ARC5 gene in plastid division; ARC5, is a protein encoded by the ARC5 gene in plastid division; ARC6, is a protein encoded by the ARC6 gene in plastid division; *BCHb*, β-ring hydroxylase enzyme; *BCHe*, ε-ring hydroxylase enzyme; *CRTISO*, carotenoid isomerase enzyme; CSR, cassava storage root; DWt, dry weight; EMBRPA, Empresa Brasileira de Pesquisa Agropecuária; FtsZ1, is a protein encoded by the FTsZ1 gene in plastid division; FtsZ2, is a protein encoded by the FtsZ2 gene in plastid division; HPLC- DAD, high performance liquid chromatography diode-array UV/VIS detector; HPLC, high performance liquid chromatography; *HYb*, beta Carotene Hydroxylase enzyme; Ln, natural logarithm; LYCb, Lycopene beta cyclase enzyme; *LYCb*, β-lycopene cyclase enzyme; *LYCe*, ε-lycopene cyclase enzyme; MinD, is a protein encoded by the MinD gene in plastid division; MinE1, is a protein encoded by the MinE1 gene in plastid division; *NXS,* neoxanthin synthase enzyme; OD, optical density; *PDS*, phytoene desaturase enzyme; qRT-PCR, quantitative reverse transcription polymerase chain reaction; SR, storage root; *VDE*, violaxanthin de-epoxidase enzyme; Z_*iso*, *Cis isomer configuration*; *ZCD*, ζ-carotene desaturase enzyme; *ZEP*, zeaxanthin epoxidase enzyme

## Additional files

Additional file 1: Figure S1. Illustration of storage root color diversity representative and tissue sampling system. Panel A –refers to close up of the cross section of the five major color groups observed in landraces collected in a center of origin and domestication of cassava in the Brazilian Amazon. Panel B –Illustrates step by step tissue sampling system for cassava storage root, Tissue sample I (Layer 1), Tissue sample II (Layer 2) and Tissue sample III (Layer 3, Layer 4, Layer 5). (TIF 64889 kb) Additional file 2: Table S1. Compiled information on germplasm documentation of the 23 collected and studied landraces. Compiled information for the 23 cassava landraces used in this document: origin, genetic background, geographical location of origin, color classes, and local utilization of cassava landraces in a center of origin and domestication of cassava in the Brazilian Amazon. W = White, IY = intense yellow, Y = yellow, PY = pale yellow, and P = Pink. (XLS 12090 kb) Additional file 3: Figure S2. HPLC_DAD reference profile for color carotenoids. Absorption spectra extracted at 485 nm wavelength reads (Panel A) used for identification and quantification of carotenoids types across 23 landraces studies. Carotenoid absorption spectrum for intense yellow root from landrace Cas64 representing the reference for carotenoid intermediates used to construct the biosynthesis pathway. Panel B refers to absorption spectrum for purified standard lycopene from tomato. Panel C refers to absorption spectrum for purified standard β- carotene from carrot. Peaks numbers refers to 1 = Neoxanthin, 2 = Violaxanthin, 3 = Zeaxanthin, 4 = Crocetin, 5 = Lutein, 6 = Antheroxanthin, 7 = Lycopene, 8 = β-cryptoxanthin, 9 = α-Zeacarotene, 10 = Neurosporene, 11 = ζ carotene, 12 = ε-zeacarotene, 13 = All trans β-carotene/Phytofluen1, 14 = 13-cis-β-carotene/Phytofluen 2, 15 = 9-cis-β-carotene/Phytofluen 2. 16 = Phytoene. (TIF 54219 kb) Additional file 4: Figure S3. HPLC_DAD reference profiles for colorless carotenoids. Absorption spectra extracted at 350 nm wavelengths reads and peaks absorption spectra for phytofluene used for comparisons across the 23 landraces studied by using two biological replications. (TIF 24310 kb) Additional file 5: Figure S4. HPLC_DAD chromatograms set1 for 9 landraces studied. Chromatograms are for carotenoids as revealed by wavelength reads at 455 nm used to identify different carotenoids types and calculation of particular carotene content across 23 landraces as compiled in Table 3 by using two biological replications. (TIF 55863 kb) Additional file 6: Figure S5. HPLC_DAD chromatograms set2 for 9 landraces studied. Chromatograms are for carotenoids as revealed by wavelength reads at 455 nm used to identify different carotenoids types and calculation of particular carotene content across 23 landraces as compiled in Table 3 by using two biological replications. (TIF 15103 kb) Additional file 7: Figure S6. HPLC_DAD chromatograms set3 for 9 landraces studied. Chromatograms are for carotenoids as revealed by wavelength reads at 455 nm used to identify different carotenoids types and calculation of particular carotene content across 23 landraces as compiled in Table 3 by using two biological replications. (TIF 15099 kb) Additional file 8: Table S2. Cassava genome annotation. Compiled information on annotation of genes coding for enzymes related to carotenoids biosynthesis and plastid division and gene sequences used for gene expression analysis with three biological replications. Proteins sequences, related to carotenoid biosynthesis, generated in this study, were registered in NCBI data base http://www.ncbi.nlm.nih.gov/protein/?term=carvalho+cassava. Protein sequences derived from cDNA sequence for microarray elements, related to plastid division, were obtained at NCBI http://www.ncbi.nlm.nih.gov/protein/?term=carvalho+cassava. Both protein sequences were annotated to cassava proteome data base   http://www.ncbi.nlm.nih.gov/protein/?term=carvalho+cassava
Additional file 9: Table S3. Information on primers used for qRT-PCR. Abbreviations are phytoene synthase (CasPSY), carotene isomerase (CasCRTISO), phytoene desaturase (CasPDS), lycopene cyclase β (CasLCYb), carotene hydroxylase β CasBCHb) and neoxanthin synthase (CasNXS) for cassava cDNA from storage root. (XLS 12142 kb)
