# Supplementary figures and images for: Natural variation in expression of genes associated with carotenoid biosynthesis and accumulation in cassava (Manihot esculenta Crantz) storage root
Source: BMC Plant Biol. 2016 Jun 10;16:133. doi: 10.1186/s12870-016-0826-0 (PMC4902922; doi:10.1186/s12870-016-0826-0)

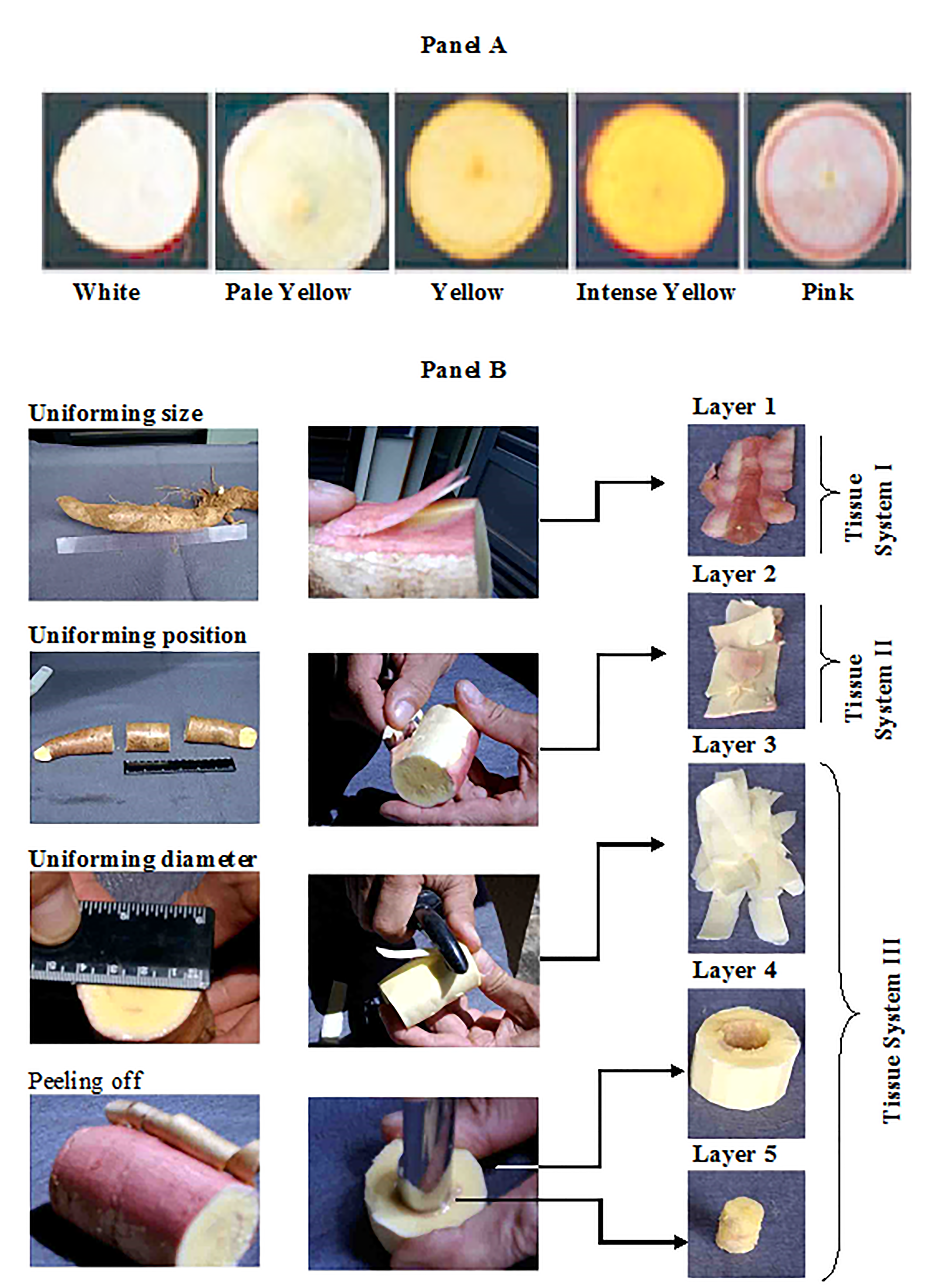

Supplement: Additional file 1: Figure S1. — Illustration of storage root color diversity representative and tissue sampling system. Panel A –refers to close up of the cross section of the five major color groups observed in landraces collected in a center of origin and domestication of cassava in the Brazilian Amazon. Panel B –Illustrates step by step tissue sampling system for cassava storage root, Tissue sample I (Layer 1), Tissue sample II (Layer 2) and Tissue sample III (Layer 3, Layer 4, Layer 5). (TIF 64889 kb) [file 12870_2016_826_MOESM1_ESM.tif]

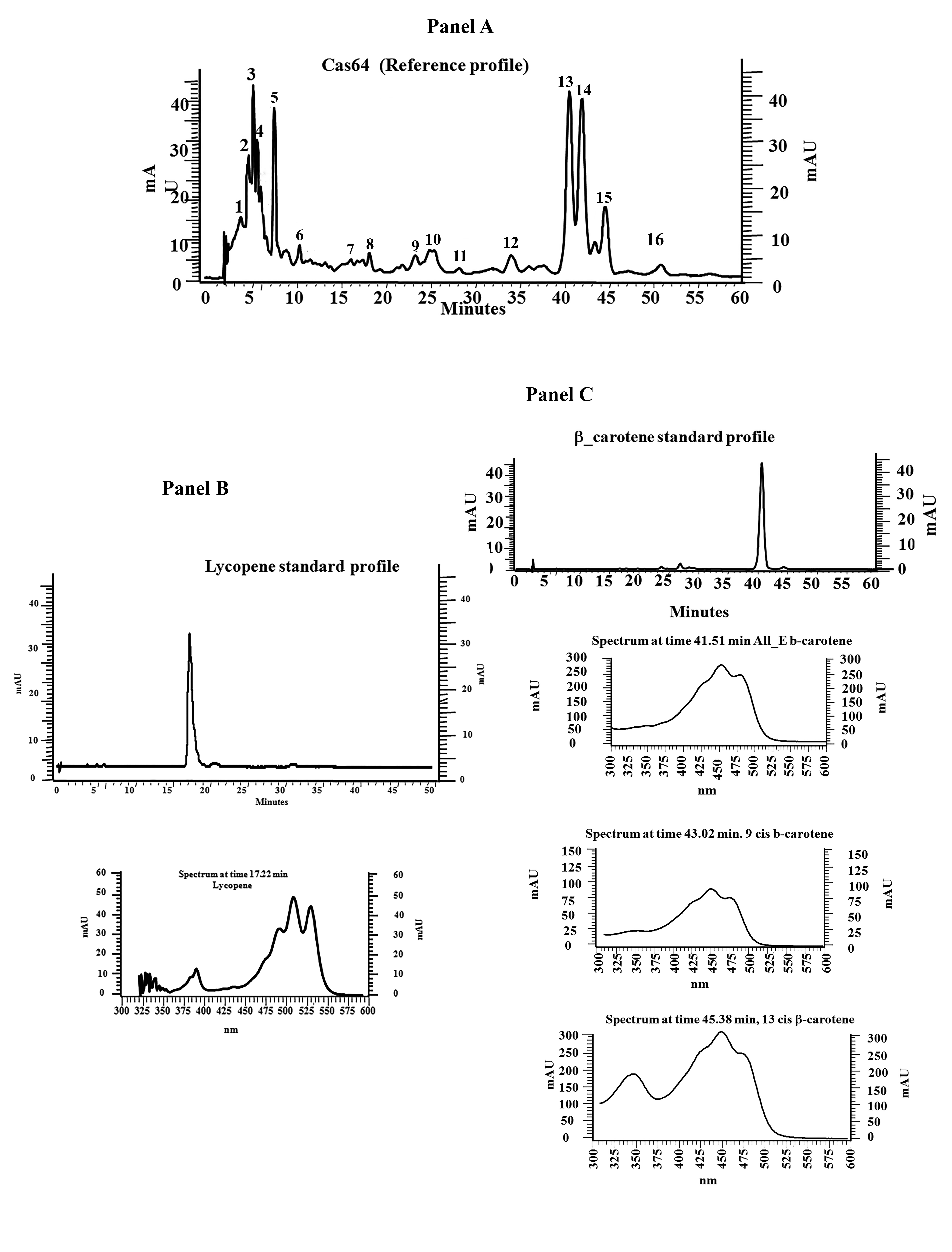

Supplement: Additional file 3: Figure S2. — HPLC_DAD reference profile for color carotenoids. Absorption spectra extracted at 485 nm wavelength reads (Panel A) used for identification and quantification of carotenoids types across 23 landraces studies. Carotenoid absorption spectrum for intense yellow root from landrace Cas64 representing the reference for carotenoid intermediates used to construct the biosynthesis pathway. Panel B refers to absorption spectrum for purified standard lycopene from tomato. Panel C refers to absorption spectrum for purified standard β- carotene from carrot. Peaks numbers refers to 1 = Neoxanthin, 2 = Violaxanthin, 3 = Zeaxanthin, 4 = Crocetin, 5 = Lutein, 6 = Antheroxanthin, 7 = Lycopene, 8 = β-cryptoxanthin, 9 = α-Zeacarotene, 10 = Neurosporene, 11 = ζ carotene, 12 = ε-zeacarotene, 13 = All trans β-carotene/Phytofluen1, 14 = 13-cis-β-carotene/Phytofluen 2, 15 = 9-cis-β-carotene/Phytofluen 2. 16 = Phytoene. (TIF 54219 kb) [file 12870_2016_826_MOESM3_ESM.tif]

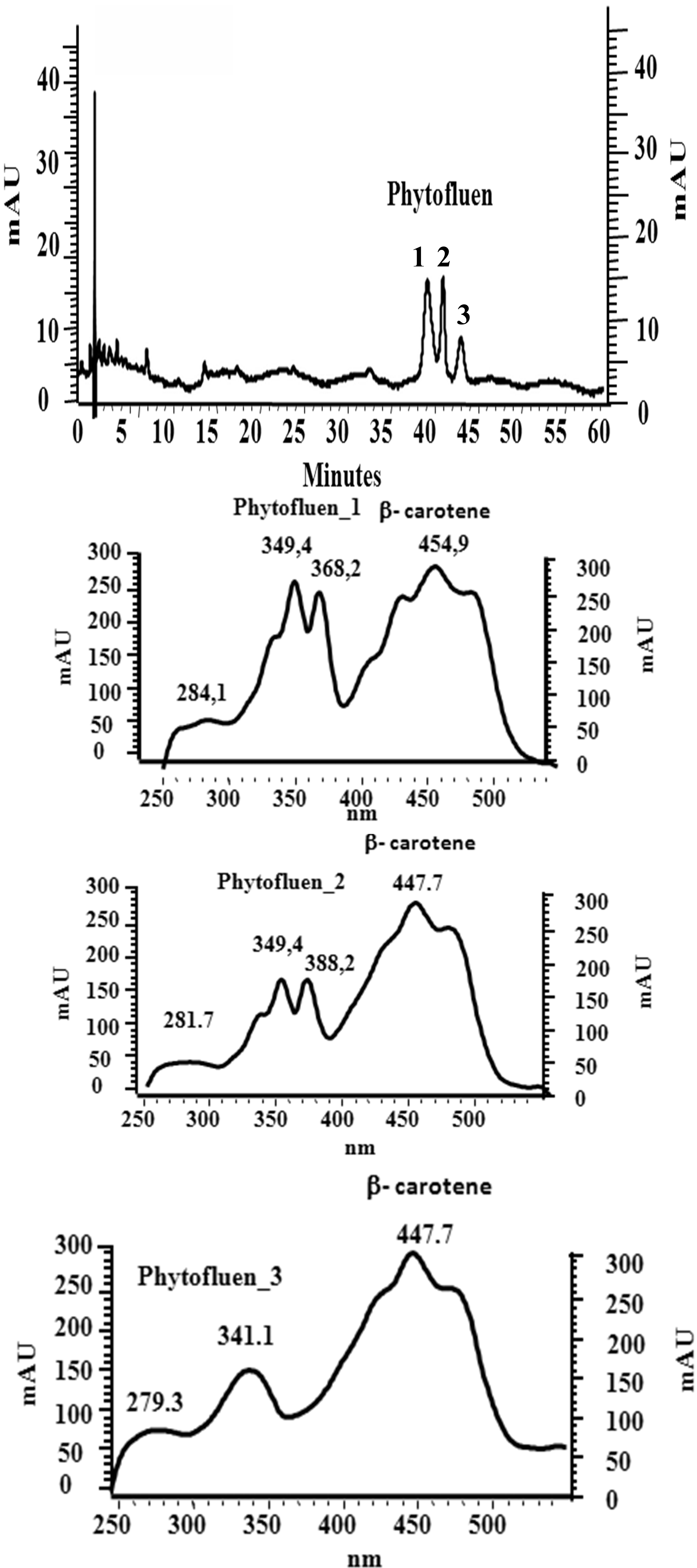

Supplement: Additional file 4: Figure S3. — HPLC_DAD reference profiles for colorless carotenoids. Absorption spectra extracted at 350 nm wavelengths reads and peaks absorption spectra for phytofluene used for comparisons across the 23 landraces studied by using two biological replications. (TIF 24310 kb) [file 12870_2016_826_MOESM4_ESM.tif]

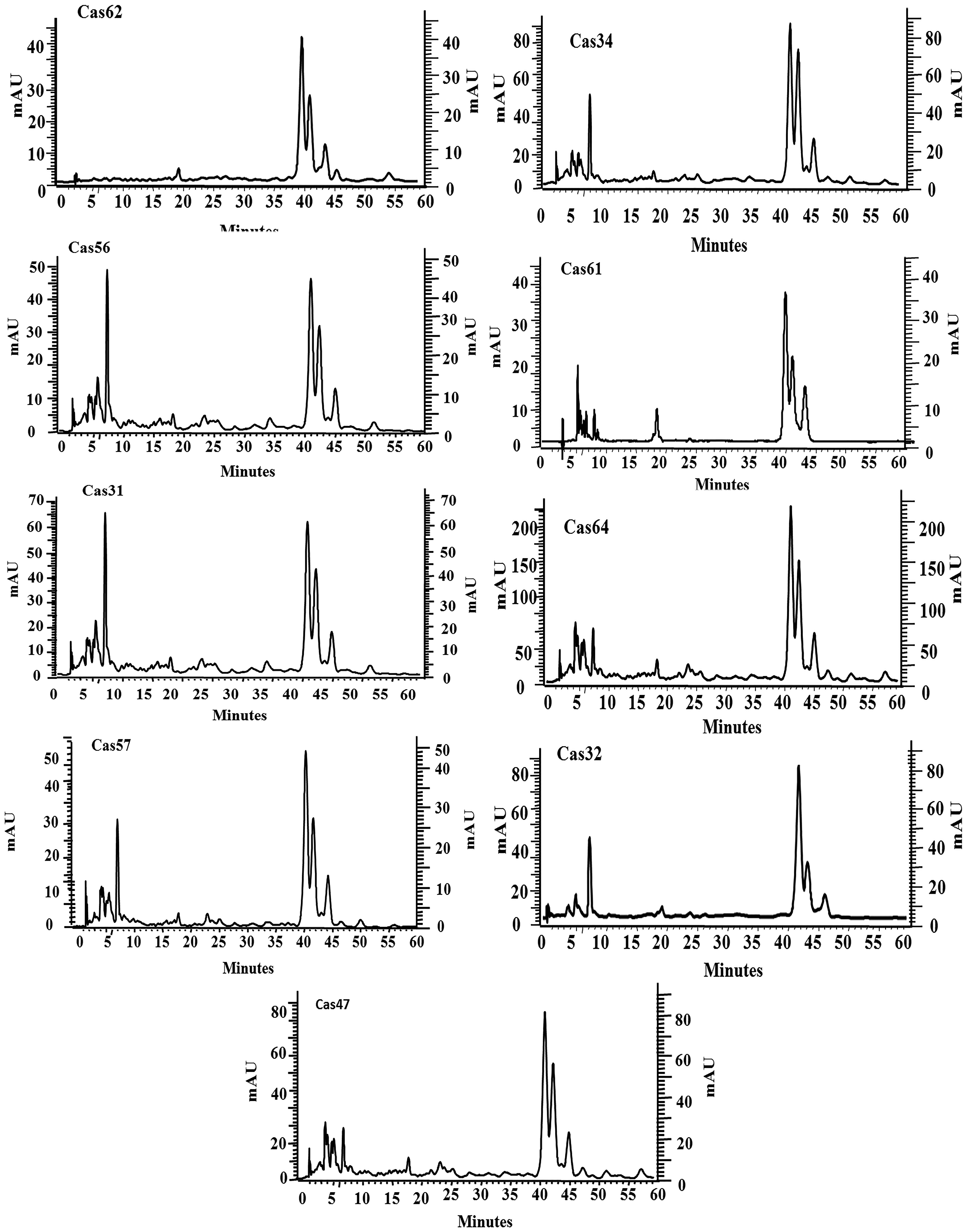

Supplement: Additional file 5: Figure S4. — HPLC_DAD chromatograms set1 for 9 landraces studied. Chromatograms are for carotenoids as revealed by wavelength reads at 455 nm used to identify different carotenoids types and calculation of particular carotene content across 23 landraces as compiled in Table 3 by using two biological replications. (TIF 55863 kb) [file 12870_2016_826_MOESM5_ESM.tif]

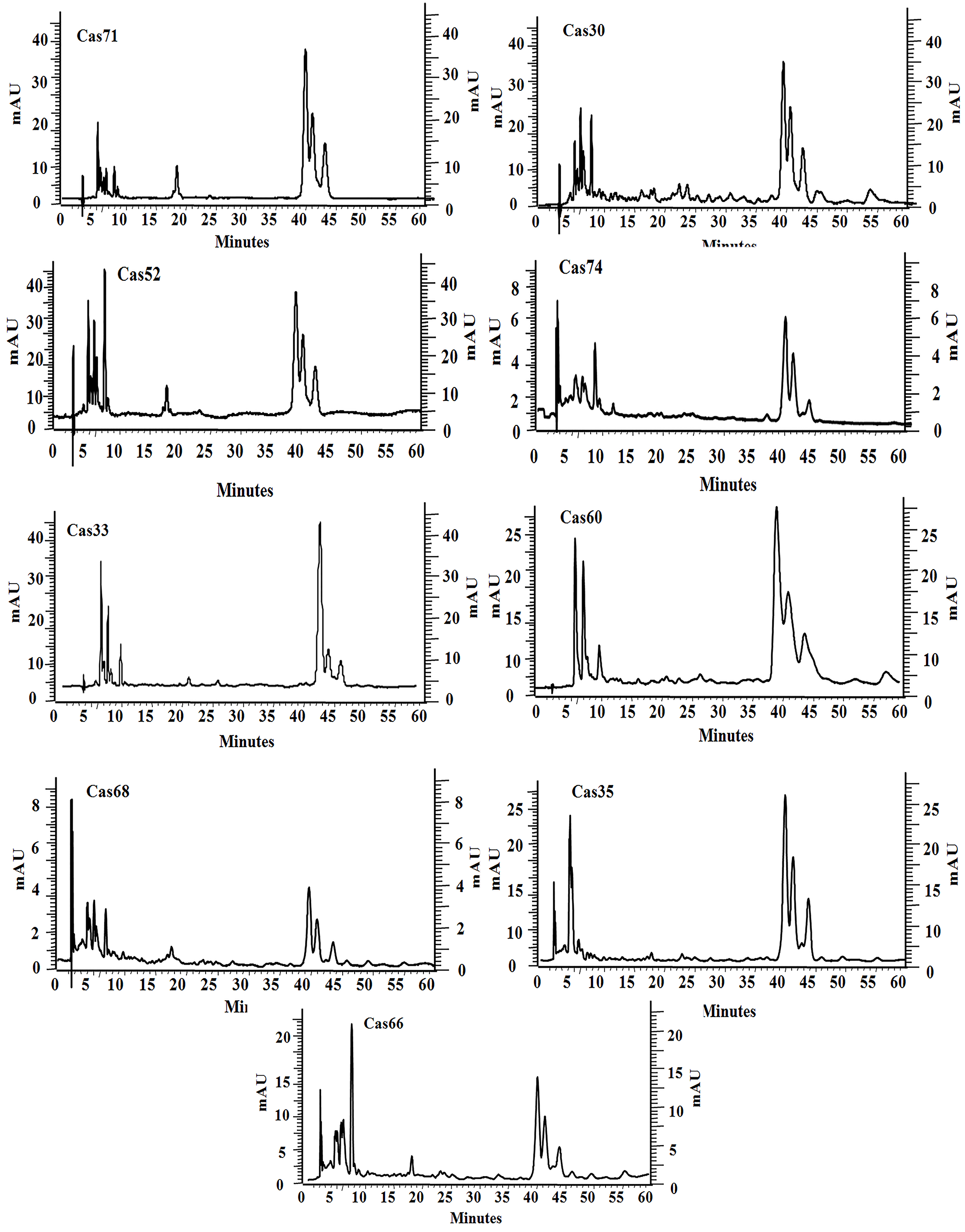

Supplement: Additional file 6: Figure S5. — HPLC_DAD chromatograms set2 for 9 landraces studied. Chromatograms are for carotenoids as revealed by wavelength reads at 455 nm used to identify different carotenoids types and calculation of particular carotene content across 23 landraces as compiled in Table 3 by using two biological replications. (TIF 15103 kb) [file 12870_2016_826_MOESM6_ESM.tif]

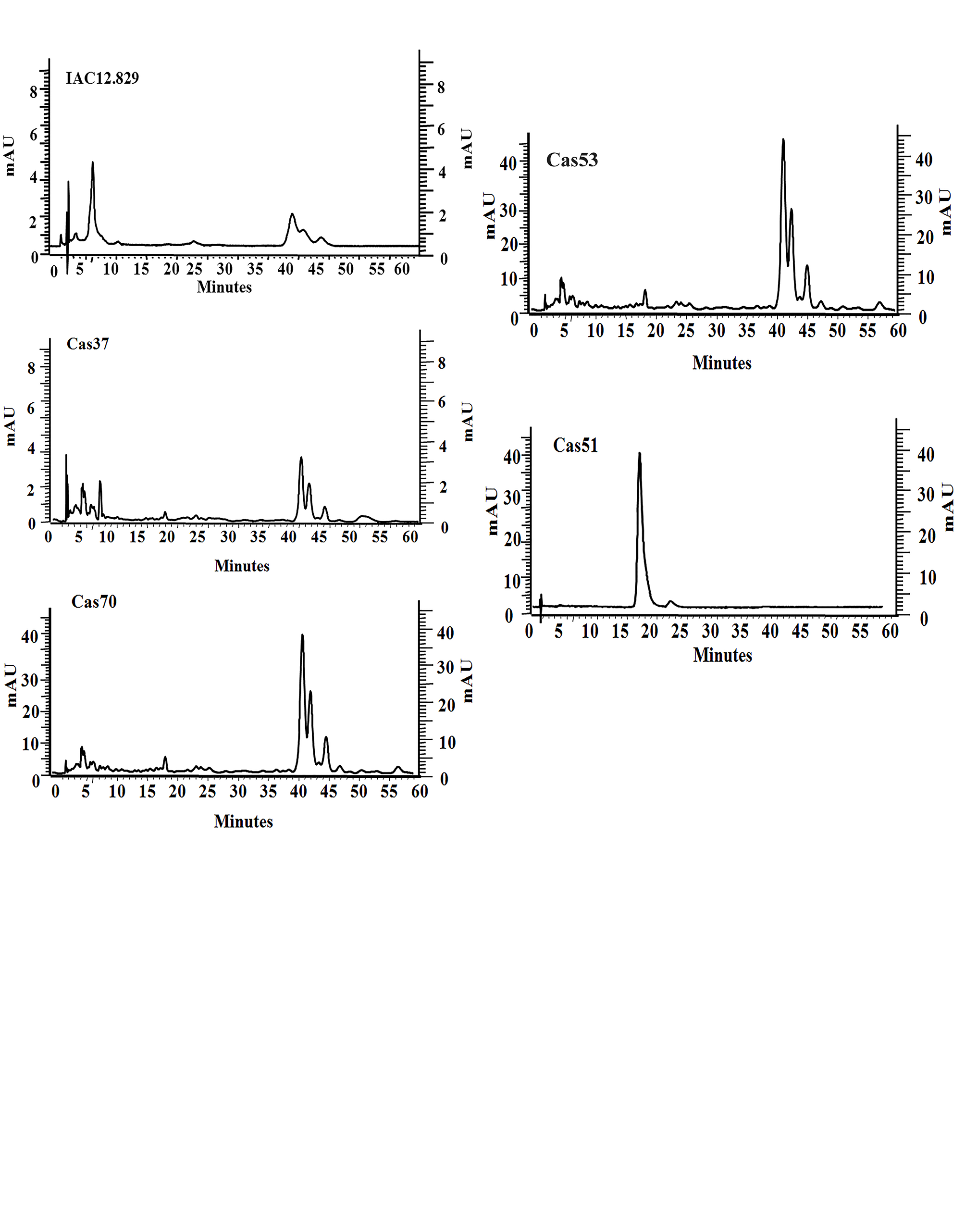

Supplement: Additional file 7: Figure S6. — HPLC_DAD chromatograms set3 for 9 landraces studied. Chromatograms are for carotenoids as revealed by wavelength reads at 455 nm used to identify different carotenoids types and calculation of particular carotene content across 23 landraces as compiled in Table 3 by using two biological replications. (TIF 15099 kb) [file 12870_2016_826_MOESM7_ESM.tif]
